# Challenges of Correlating pH Change with Relief of Clinical Symptoms in Gastro Esophageal Reflux Disease: A Phase III, Randomized Study of Zegerid versus Losec

**DOI:** 10.1371/journal.pone.0116308

**Published:** 2015-02-23

**Authors:** Dave Walker, Richard Ng Kwet Shing, Deborah Jones, Hans-Jurgen Gruss, Jarosław Reguła

**Affiliations:** 1 Norgine Ltd, Uxbridge, United Kingdom; 2 Medical and Safety Services, ICON Clinical Research, Eastleigh, United Kingdom; 3 Department of Gastroenterology, Institute of Oncology and Medical Centre for Postgraduate Education, Warsaw, Poland; Weill Cornell Medical College Qatar, QATAR

## Abstract

**Background:**

Zegerid (on demand immediate-release omeprazole and sodium bicarbonate combination therapy) has demonstrated earlier absorption and more rapid pH change compared with Losec (standard enteric coated omeprazole), suggesting more rapid clinical relief of heartburn. This Phase III, multicenter, double-blind, double-dummy, randomized study assessed the clinical superiority of Zegerid versus Losec for rapid relief of heartburn associated with gastro-esophageal reflux disease (GERD).

**Methods:**

Patients with a history of frequent (2 3 days/week) uncomplicated GERD, were randomized to receive Zegerid (20mg) or Losec (20mg) with corresponding placebo. Study medication was self-administered on the first episode of heartburn, and could be taken for up to 3 days within a 14 day study period. Heartburn severity was self assessed up to 180 minutes post dose (9 point Likert scale). Primary endpoint was median time to sustained response (≥3 point reduction in heartburn severity for ≥45 minutes).

**Results:**

Of patients randomized to Zegerid (N=122) or Losec (N=117), 228/239 had recorded ≥1 evaluable heartburn episodes and were included in the modified intent-to-treat population. No significant between-group differences were observed for median time to sustained response (60.0 vs. 52.2 minutes, Zegerid [N=117] and Losec [N=111], respectively), sustained partial response (both, 37.5 minutes) and sustained total relief (both, 105 minutes). Significantly more patients treated with Zegerid reached sustained total relief within 0–30 minutes post dose in all analysis sets (p<0.05). Both treatments were well tolerated and did not raise any safety concerns.

**Conclusions:**

Superiority of Zegerid over Losec for rapid heartburn relief was not demonstrated; both treatments were equally effective however the rapid onset of action of Losec was unexpected. Factors, including aspects of study design may have contributed to this. This study supports previously reported difficulty in correlating intra-gastric pH change with clinical effect in GERD therapy, highlighting the significance of several technical considerations for studies of this type.

**Trial registration:**

ClinicalTrials.gov NCT01493089

## Background

Gastro-esophageal reflux disease (GERD) has been defined as ‘a condition which develops when the reflux of stomach contents causes troublesome symptoms and/or complications’[1]. GERD is a common condition, particularly in the Western world, where the prevalence has been estimated to range between 10% and 20% using a definition of at least weekly heartburn and/or acid regurgitation[2]. Population studies in Spain, Sweden and the United Kingdom (UK) reported a prevalence of frequent (at least weekly) heartburn and/or acid regurgitation of 9.8%, 16.7% and 18%, respectively[2–5].

Management of GERD focuses on symptom control and, as the severity of GERD varies significantly between patients, it should be individualized. Lifestyle modifications, including weight loss for overweight/obese patients and avoiding specific foods, may improve GERD outcomes[6]. Initial management for patients with uncomplicated heartburn is maintenance anti-secretory therapy with proton pump inhibitors (PPIs) or histamine_2_ receptor antagonists (H_2_RAs). Anti-reflux surgery is recommended when a patient with GERD is responsive to, but intolerant of, acid suppressive therapy, or when troublesome symptoms persist despite PPI therapy[6].

On-demand, patient-driven therapy with PPIs may provide cost-effective, convenient, successful treatment for GERD, other than for severe esophagitis[7]. Importantly, on-demand therapy differs from intermittent therapy, which requires predefined intermittent episodes of continuous therapy followed by discontinuation until symptoms recur. A systematic review encompassing 17 studies concluded that on-demand therapy with currently available PPIs is effective in the long-term management of GERD, excluding erosive esophagitis[8].

**Fig 1:**
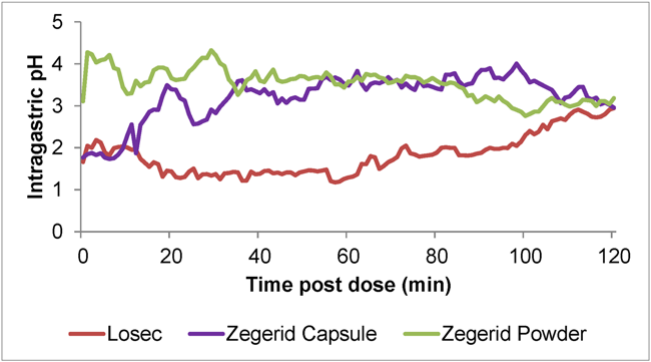
Mean intragastric pH, PP population, Unpublished Phase I study.

Zegerid (Santarus Inc, [now Salix], USA) has been developed for improved, rapid symptom relief of the symptoms of GERD as on-demand therapy. The two active ingredients in Zegerid are omeprazole, an established PPI therapy for GERD, and sodium bicarbonate, known to neutralize gastric acid. Most oral PPI preparations, including omeprazole, are enteric-coated in order to avoid rapid degradation of the drugs in the acidic conditions of the stomach; however this results in delayed-release characteristics. Zegerid contains an immediate-release preparation of omeprazole that does not require an enteric coating since the combination with sodium bicarbonate acts as a buffer to protect omeprazole from gastric acid degradation. The antacid sodium bicarbonate also provides rapid neutralization of gastric acid and, therefore, may provide faster relief of symptoms independent of the accelerated effect on omeprazole absorption. Preliminary pharmacokinetic (PK) and pH data from a randomized Phase I study in healthy volunteers suggested an onset benefit for this new combination formulation of omeprazole with sodium bicarbonate (Norgine, data on file). The Phase I study investigated the relative bioavailability and pharmacodynamic profiles of Zegerid (immediate-release omeprazole/sodium bicarbonate) 20mg capsule and Zegerid powder for oral suspension (Zegerid suspension) versus a comparator arm, Losec (enteric-coated omeprazole capsule, AstraZeneca, UK) 20mg. Intragastric pH rose more rapidly after treatment with both Zegerid formulations than after Losec in the Per Protocol (PP) and Intention-to-Treat (ITT) populations (Fig. 1). Zegerid capsules demonstrated a faster time to maximum plasma concentration (Tmax) compared with Losec (0.50 vs. 1.38 hours, p = 0.0001) and Zegerid suspension showed a superior Tmax compared with Losec (0.38 hours versus 1.38 hours, p<0.001). Based on these pH data, it was proposed that both Zegerid formulations may provide more rapid clinical relief of heartburn symptoms associated with GERD than delayed-release omeprazole.

The previously held conventional theory was that heartburn symptoms are secondary to acid reflux[9–13]. Therefore, pH was an acceptable surrogate marker for heartburn, with an increase of esophageal pH linked directly to symptom relief[14,15]. However, it has been demonstrated that ambulatory esophageal pH monitoring in patients refractory to PPI therapy is predominantly normal[16,17]. It is now recognized that non-acid reflux can produce heartburn symptoms, and may contribute to the persistence of symptoms in patients on PPI therapy[18]. Indeed, simultaneous intraesophageal impedance and pH measurement in patients with persistent symptoms despite acid-suppressive therapy demonstrated that symptoms were associated with non-acid reflux in around a third of patients[19–21]. Other constituents of gastric contents may induce esophageal symptoms[22,23], with bile and pepsin both identified as contributing to reflux episodes[23,24].

Due to the contribution of other mechanisms to symptomatic reflux episodes, regulatory authorities no longer accept intragastric pH as a surrogate marker for heartburn. The European Medicines Agency (EMA) ‘Guideline on the evaluation of drugs for the treatment of gastro-esophageal reflux disease’ requires demonstration of symptomatic relief in clinical trials and states that for the later phases of drug development (Phase IIb and Phase III), symptom-based evaluation is required as the primary basis of proof of efficacy[25]. Thus, demonstration of a rise in intragastric pH is not sufficient to demonstrate efficacy to the satisfaction of European Union (EU) Regulators. Consequently, the key objective of this patient-outcomes trial was to assess the clinical superiority of Zegerid versus Losec in the rapid relief of heartburn.

## Methods

The protocol for this trial and supporting CONSORT checklist are available as supporting information; see S1 Checklist and S1 Protocol.

### Study design

This Phase III, multicenter, double-blind, double-dummy, randomized study assessed the superiority of Zegerid suspension 20mg versus Losec 20mg as on-demand therapy in the rapid relief of heartburn associated with GERD. The study was performed in 39 study sites in six EU countries (Bulgaria, Czech Republic, Hungary, Poland, Romania and the UK). Each of the 39 study sites was blinded and identified only by a site identifier number. In Bulgaria, sites 101, 102, 103, 104 and 106 recruited 7, 5, 8, 2 and 7 patients, respectively. In the Czech Republic, sites 201, 202, 203, 206 and 207 recruited 8, 6, 4, 10 and 3 patients, respectively. In Hungary, sites 301, 302, 303, 304, 305, 306, and 307 recruited 5, 5, 2, 3, 7, 4 and 7 patients respectively. In Poland, sites 401, 402, 403, 404, 405, 406, 407, 408, 409, 410, 411, 412, 413, and 414 recruited 6, 2, 9, 3, 9, 6, 11, 6, 8, 2, 8, 3, 7 and 12 patients, respectively. In Romania, sites 501, 502, 503, 504, 505 and 506 recruited 7, 4, 4, 11, 3 and 4 patients, respectively. In the UK, sites 601 and 602 recruited 4 and 6 patients, respectively. Recruitment started on 26 April 2011 and last patient last visit was on 8th September 2011. Enrolled patients entered a 7-day baseline symptom-assessment screening period, during which they recorded the severity of daily episodes of heartburn using a 9-point Likert severity scale and an electronic diary (e-diary). The only antacid medication permitted during this screening period was the specified rescue medication (Gaviscon; chewable tablets; Reckitt Benckiser Group, UK). Eligible subjects were then randomized 1:1 to receive Zegerid suspension 20mg or Losec 20mg in a double-dummy design during the 14-day study period and were followed-up on Day 15. The randomization list, available via Interactive Web Response System (IWRS), was produced by an independent statistician (Premier Research Group Ltd.), who was not involved in the conduct or analysis of the study, using the SAS system for Windows (SAS Institute Inc).

### Ethics statement

The study was conducted in compliance with the Declaration of Helsinki and Good Clinical Practice guidelines. The protocol (S1 Protocol) was approved by the relevant Independent Ethics Committees in Bulgaria, Czech Republic, Hungary, Poland, Romania and the UK between January 2011 and June 2011, prior to each center’s initiation. Written informed consent was received from all subjects prior to enrolment into the study. This study was registered on EudraCT (2010–022082–10; https://www.clinicaltrialsregister.eu/ctr-search/search?query=eudract_number:2010-022082-10) and on ClinicalTrials.gov (clinical trial registration number NCT01493089; http://clinicaltrials.gov/ct2/show/NCT01493089?term=NCT01493089&rank=1). This study was submitted to ClinicalTrials.gov for registration prior to study commencement, however due to an administrative delay it was made publically available on the registry after patient enrolment had begun. At the time of initial submission to ClinicalTrials.gov, only the site in Poland, under the supervision of the Principal Investigator had been confirmed for inclusion in the study, and was therefore listed in the study entry. The authors confirm that all ongoing and related trials for this drug/intervention are registered. The authors and this clinical study followed CONSORT guidelines for the reporting of randomized trials.

### Patients

Patients were male or female, 18–75 years of age, with a history of frequent episodes of heartburn associated with GERD for at least 2–3 days per week during the 2–4 weeks prior to screening, and had responded to standard PPI therapy in the past 12 months. Patients had not taken on-demand PPI therapy for >3 consecutive days within 4 weeks prior to screening and had recorded at least one evaluable episode of heartburn on 2 separate days at level 4 or higher on the 9-point Likert severity scale during the screening period. Competent use and completion of the e-diary during the screening period was also required. Females of childbearing potential had to employ an adequate method of birth control.

Patients were excluded if they had taken any medication for the purpose of the eradication of *Helicobacter pylori* (*H. pylori*), systemic glucocorticoids or non-steroidal anti-inflammatory drugs (except enteric-coated aspirin) during the 28 days prior to the study. Other excluded drugs were concomitant medications that rely on the presence of gastric acid for optimal bioavailability; psychoactive medication in the past 6 months and during the study; and other specified medications taken within 2 weeks of the first dose of study medication or for concurrent therapy. Patients were also excluded due to previous acid-lowering surgery or other surgery of the esophagus or upper gastrointestinal tract; history of co-existing disease affecting the esophagus and had incomplete healing of erosions following standard PPI therapy within the last 3 months; history of active gastric or duodenal ulcers within the last 3 months; or had acute upper gastrointestinal bleeding within the last 6 months. Severe renal or hepatic insufficiency, clinically significant laboratory abnormality or disease, and known hypersensitivity to omeprazole resulted in exclusion.

### Study treatment

Following the screening period, eligible patients were randomized 1:1 using a double-dummy design to receive either Zegerid suspension 20mg with over-encapsulated placebo capsule, or Losec 20mg over-encapsulated capsule with placebo suspension.

Zegerid oral suspension contained omeprazole, sodium bicarbonate, xylitol, sucrose, sucralose, xanthan gum and flavorings (peppermint and peach). The flavorings were added to mask the taste of the sodium bicarbonate. The Zegerid-matching placebo was powder for oral suspension. Losec over-encapsulated capsule contained omeprazole, mannitol, hyprolose, cellulose microcrystalline, anhydrous lactose, sodium lauril sulphate, disodium hydrogen phosphate dihydrate, hypromellose, methacrylic acid copolymer, macrogol, colors E171 and E172, gelatin and magnesium stearate. The Losec-matching placebo was over-encapsulated capsule. The placebo contained sucralose, xanthan gum, sodium chloride, maltodextrin, xylitol, peppermint (flavor), artificial peach powder (flavor), starch (corn) and sugar powder.

Patients were to take a maximum of one pack of randomized study medication (1 dose each of suspension and capsule) per day, for a maximum of 3 days over the 14-day randomized study period. Study medication was self-administered when the first episode of heartburn (level 4 or higher on the 9-point Likert scale) was experienced for that day. Each episode of heartburn was recorded using the e-diary.

Rescue medication (Gaviscon) was provided for use during the screening period (up to 3 tablets per day) and study period (up to 2 tablets per day, at least 3 hours after taking study medication or any time outside the 3 days of taking study medication).

### Study assessments and endpoints

A 9-point Likert scale (‘0’ for no heartburn to ‘8’ for very severe heartburn) was used for self-assessment of symptoms, and scores were recorded using an e-diary. During the 7-day screening period, patients recorded the occurrence and severity of daily episodes of heartburn. Between 06:00 and 22:00 h daily during the study period, patients recorded the severity of each heartburn episode for which randomized study medication and placebo were taken, pre-dose and at 15, 30, 45, 60, 75, 90, 105, 120, 150 and 180 minutes post-dose. After 22:00 h, patients were not to take study medication if all the time points up to 180 minutes could not be recorded, for example, if the patient was not prepared to stay awake for 180 minutes if woken by an episode of GERD (rescue medication could be taken instead).

The primary endpoint was the median time to sustained response, defined as a reduction in severity of heartburn associated with GERD by 3 points or more on the 9-point Likert severity scale, which was sustained for at least 45 minutes. Key secondary efficacy assessments (which were sustained for 45 minutes or more) were the median time to sustained partial response and sustained total relief, and the proportion of patients who achieved sustained response, sustained partial response or sustained total relief by 45, 60 and 90 minutes. Using the Likert severity scale, partial response was defined as a reduction in severity of heartburn by 2 points or more, and total relief was defined as ‘0’ severity (no heartburn).

Other secondary efficacy assessments included the severity of heartburn associated with GERD and change in severity from pre-dose at all timepoints; the proportion of patients who had achieved sustained response, partial response or total relief at all timepoints; and the use of Gaviscon (rescue medication) over the 14-day randomized study period.

Safety assessments included monitoring of adverse events (AEs), laboratory parameters (hematology, biochemistry and urinalysis), vital signs, physical examination and concomitant medication.

### Statistical analysis

In order to determine the sample size, a log-rank test was employed. It was assumed from Phase I PK and pH data that conservative estimates (compared to previously reported values of 30 minutes and 102 minutes respectively) for the median time to response for Zegerid and Losec were 45 and 75 minutes, respectively. Therefore, with 90% power and a two-sided level of significance of 0.05 (5%), 97 patients were required per treatment group.

Four analysis populations were defined. The Safety Set (ITT) consisted of all patients who received at least one dose of randomized study medication during the course of the study. The modified ITT (mITT) population consisted of all patients who had recorded data for at least one evaluable episode of heartburn. The PP set excluded patients who violated any inclusion/exclusion criterion, or with major protocol violations, and the PP2 set also excluded patients who received study drug that was stored below the recommended storage temperature.

The primary statistical hypothesis, evaluated using a confirmatory test, was that there was no difference in the time to sustained relief in the two treatment groups. Differences between the two treatment groups were tested at a two-sided significance level of 0.05 using a Cox regression model, including a country effect. All efficacy parameters were defined using the median of the one to three heartburn severity scores at each time point with the patient as the unit of analysis. Each patient could have up to three evaluable episodes of heartburn and in the primary analysis the average (median) of severity was derived for each time point over the evaluable episodes before any analysis was performed. Secondary endpoints were analyzed using Kaplan-Meier analysis and chi-squared tests. All efficacy analyses were performed using the mITT, PP and PP2 sets.

While all data were collected prospectively, an additional exploratory efficacy analysis was planned and conducted after locking the database and unblinding the data. In the exploratory analysis, the episode was the unit of analysis and each GERD episode was treated as an individual data point, stratified by severity and time. The primary and key secondary efficacy parameters, and the proportion of episodes that had achieved sustained response, partial response or total relief at all timepoints were included in the exploratory analysis.

## Results

### Patients (baseline characteristics and disposition)

**Fig 2:**
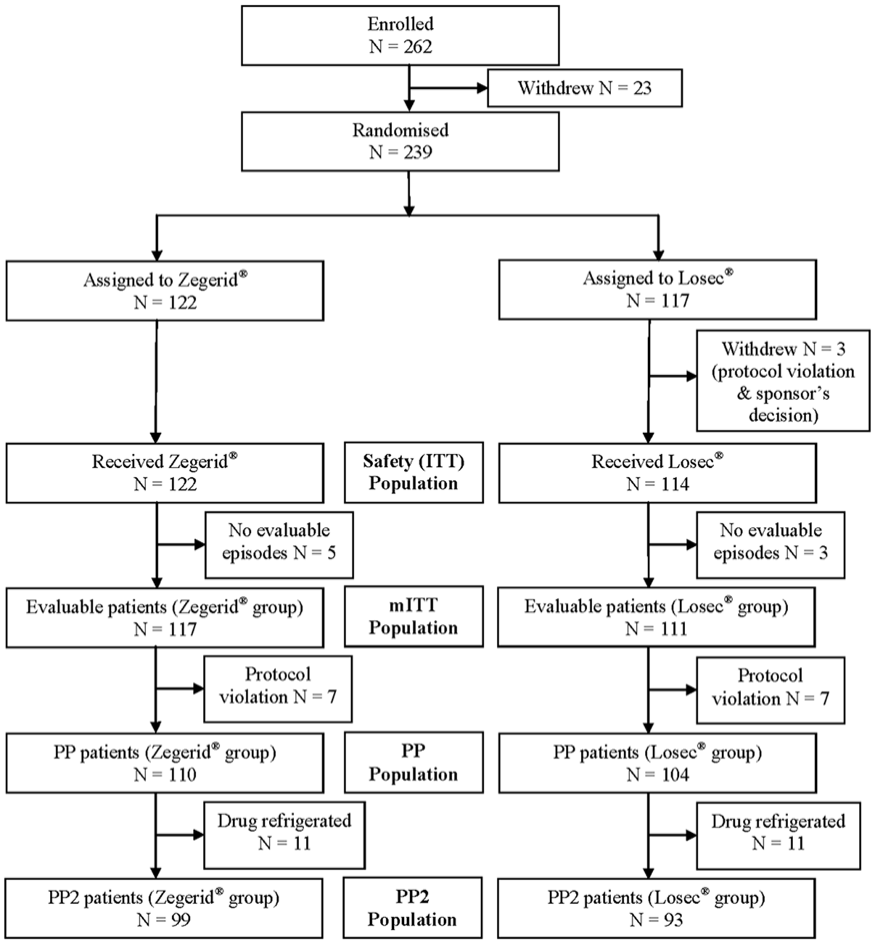
Study flow diagram.

Between 14 April 2011 and 08 September 2011, 262 patients were enrolled and 239 randomized to receive Zegerid (N = 122) or Losec (N = 117). Baseline characteristics and demographics were generally similar in the two treatment groups (Table 1). Except for one non-Caucasian patient in the Zegerid group, all patients were Caucasian (99.6%), and all patients (100%) had a history of gastrointestinal disorders. Fig. 2 summarizes participant flow through the study. One patient withdrew due to a serious AE (SAE; cholecystitis acute) prior to randomization; however, no patients withdrew due to treatment-emergent AEs (TEAEs).

**Table 1:** Baseline patient characteristics (safety, ITT population).

Variable		20mg Zegerid (N = 122)	20mg Losec (N = 114)
Gender, n (%)	Female	62 (50.8)	66 (57.9)
	Male	60 (49.2)	48 (42.1)
**Demographics (mean±SD [range])**			
Age (years)		45.0±15.43 (18–74)	44.4±13.91 (20–75)
Weight (kg)		77.26±14.379 (43.0–125.0)	75.67±15.807 (48.0–110.0)
Height (cm)		170.9±9.46 (148–191)	169.8±9.67 (152–197)
BMI (kg/m^2^)		26.36±3.962 (18.4–38.1)	26.25±5.188 (17.0–43.1)

Abbreviations: ITT = intention-to-treat, n = number of patients, SD = standard deviation, BMI = body mass index.

### Efficacy

**Fig 3:**
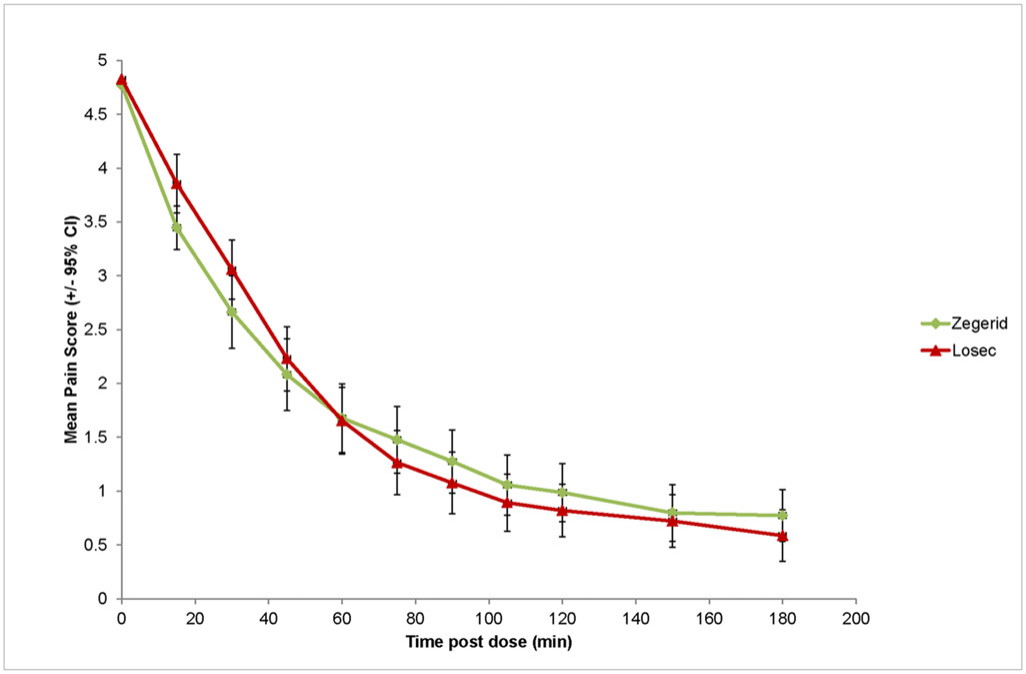
Severity of heartburn by time (mITT set, N = 228).

**Fig 4:**
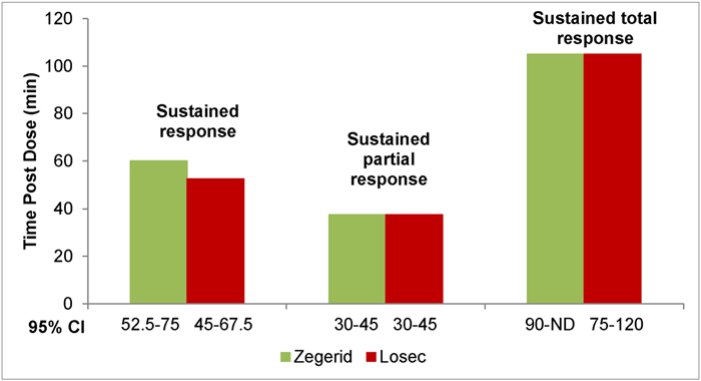
Median time to sustained response, sustained partial response and sustained total relief (mITT set, N = 228; p = 0.5363).

Both Zegerid and Losec were effective at reducing the severity of heartburn associated with GERD (Fig. 3). However, the superiority of Zegerid versus Losec in the rapid relief of heartburn was not demonstrated for the majority of primary or secondary endpoints. The primary endpoint, median time to sustained response, was 60 minutes in the Zegerid group and 52.2 minutes in the Losec group. Of the 117 Zegerid and 111 Losec patients in the mITT population, sustained response was achieved in 88 (75.2%) Zegerid and 90 (81.1%) Losec patients (Fig. 4). No statistically significant difference between the treatment groups was demonstrated.

**Fig 5:**
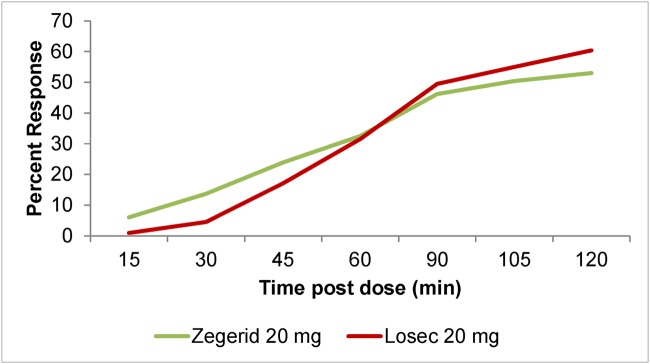
Time to sustained total relief (mITT set, N = 228; p = 0.4570).

For the secondary endpoints, the median time to sustained partial response was 37.5 minutes in both the Zegerid and Losec patient groups. In the mITT population, sustained partial response was achieved in 109 (93.2%) Zegerid and 98 (88.3%) Losec patients. Similarly, the median time to sustained total response was 105 minutes in both groups (Zegerid, 62 patients [53.0%]; Losec, 67 patients [60.4%]; Fig. 5). The proportion of patients who achieved sustained response, partial response or total relief between 45 and 180 minutes showed no significant difference between the treatment groups in any analysis set.

There was a trend towards more patients with sustained response by 30 minutes in the Zegerid group than in the Losec group (Table 2). Additionally, a greater proportion of patients had sustained total relief by 30 minutes in the Zegerid group than in the Losec group in all analysis sets (13.7% vs. 4.5%, p = 0.0199 mITT, 13.6% vs. 4.8%, p = 0.0315 PP, 14.1% vs. 4.3%, p = 0.0267 PP2). After taking study medication, a faster decrease in the severity of heartburn was observed in the Zegerid group than the Losec group at 15 (-27.3% versus-18.6%), 30 (-43.9% versus-34.7%) and 45 minutes (-55.4% versus-51.8%). After 60 minutes, however, the decrease was similar in both treatment groups (~64%). After 75–180 minutes, the Losec group showed a faster decrease in severity (up to-86.0% compared with-83.6% in the Zegerid group).

**Table 2:** Proportion of patients with sustained response by 30 minutes.

	Zegerid	Losec	P value
**mITT Set**	**(N = 117)**	**(N = 111)**	
Responder Rate (%)	35.0	25.2	0.0891
95% CI of Rate (%)	26.5–44.4	17.5–34.4	0.0891
**PP Set**	**(N = 110)**	**(N = 104)**	
Responder Rate (%) (%)Responders	35.5	25.0	0.0900
95% CI of Rate (%)	26.6–45.1	17.0–34.4	0.0900
**PP2 Set**	**(N = 99)**	**(N = 93)**	
Responder Rate (%) (%)Responders	38.4	24.7	0.0351
95% CI of Rate (%)	28.8–48.7	16.4–34.8	0.0351

Abbreviations: CI = confidence interval, ITT = intention-to-treat, n = number of patients, mITT = modified ITT, PP = per protocol.

Regarding best overall sustained response, the responder rate was similar in both treatment groups (22.2% vs. 20.7%). The percentage of patients with partial response in the Zegerid group compared with the Losec group was 17.9% vs. 7.2%, respectively. The percentage of non-responders and patients with total relief, respectively in the Losec group were 11.7% and 60.4% compared with 6.8% and 53.0% in the Zegerid group.

No advantage for Zegerid over Losec was observed for the use of rescue medication. During screening, the mean number of Gaviscon tablets taken per day was 1.20 and 1.17 in the Zegerid and Losec groups, respectively. During treatment, the mean number of Gaviscon tablets taken per day was 0.46 and 0.33 in the Zegerid and Losec groups, respectively. The mean interval between taking study medication and the next use of medication (study medication or Gaviscon) was 48.61 and 64.27 hours in the Zegerid and Losec groups, respectively.

In the exploratory analysis, no statistically significant difference between the treatment groups was demonstrated for the primary or secondary endpoints or for any stratified population, by severity or time.

### Safety

The majority of patients took the maximum 3 doses in both the Zegerid and Losec groups (102 [83.6%] and 95 [83.3%] patients, respectively). Overall, no safety concerns were raised with generally similar safety profiles between the treatment groups. In the Zegerid group, 4 (3.3%) patients reported 6 TEAEs all of which were gastrointestinal disorders, including one event each of nausea and abdominal pain upper, and two events each of abdominal distension and flatulence. In the Losec group, 4 (3.5%) patients reported 5 TEAEs, consisting of one event each of nausea, influenza-like illness, paronychia, headache and epistaxis. All TEAEs were classified as mild, except influenza-like illness and paronychia, which were moderate in severity. There were three drug-related TEAEs, two of abdominal distension and one of nausea, in the Zegerid group. All TEAEs resolved except for the event of paronychia in the Losec group, which was ongoing at the end of the study.

No deaths, treatment-emergent SAEs, AEs leading to withdrawal, AEs requiring an intervention or laboratory TEAEs were reported during the study. One SAE (cholecystitis acute) led to the withdrawal of a single patient prior to randomization.

## Discussion

This study was conducted to assess the time to sustained symptom relief in patients suffering from heartburn associated with GERD treated with Zegerid compared with Losec. Zegerid was effective at relieving the symptoms of GERD, it was well tolerated and no safety concerns were raised. In terms of patient outcomes, however, the superiority of Zegerid was not demonstrated. No significant differences between the Zegerid and Losec groups were observed in the times to sustained response, sustained partial response and sustained total relief. The proportion of patients reaching sustained total relief over time was significantly higher in the Zegerid group after 30 minutes in all analysis sets; however, no other significant differences were observed. For best overall sustained response, the sustained responder rate was similar in both treatment groups. While the Zegerid group showed a higher percentage of patients with sustained partial response, the Losec group showed more non-responders and patients reaching sustained total relief. The severity of heartburn was similar in both treatment groups 180 minutes after medication intake.

Zegerid is a novel, fixed combination of omeprazole and sodium bicarbonate. Omeprazole is an established therapy for GERD and belongs to the class of anti-secretory PPIs, which are substituted benzimidazole derivatives that selectively and irreversibly inhibit the H^+^/K^+^ adenosine triphosphatase (ATPase) enzyme system at the secretory surface of parietal cells[26,27]. Proton pump inhibition is greatest in those parietal cells that are actively secreting acid; therefore, the anti-secretory effect of PPIs is optimal after a meal, when the parietal cell and its proton pumps are maximally stimulated[28]. Consequently, gastric pH control with PPIs is affected by dose-timing relative to a meal[15]. In Zegerid, the sodium bicarbonate provides rapid neutralization of gastric acid which protects omeprazole from gastric acid degradation. The PPI can, therefore, be rapidly absorbed from the stomach allowing earlier onset of action than delayed-release preparations such as Losec, which are intended to reach the intestine before being broken down and absorbed.

Previous Phase I PK and pH studies clearly demonstrated that immediate-release omeprazole/sodium bicarbonate (Zegerid) increases intragastric pH more rapidly than delayed-release omeprazole (Losec). Fig. 1 illustrates that Zegerid suspension increased intragastric pH > 4 after approximately 5 minutes post-dose, whereas intragastric pH remained below 3 up to 120 minutes after Losec administration. Based on the Phase I studies, it was proposed that Zegerid may provide more rapid clinical relief of GERD symptoms than Losec. However, the results of this study do not correlate clinical outcomes with Phase I PK and pH evidence.

Although few clinical studies have directly examined the relationship between intra-gastric pH control and symptom relief, previous studies have met with a similar challenge to that of the current study, reflecting the difficulty in correlating intra-gastric pH with clinical effect in the treatment of GERD[15,29,30]. For example, the acid-suppressing agent AZD0865 demonstrated a rapid onset of acid suppression, however, when compared with esomeprazole this effect did not translate into more rapid clinical benefit[29,30]. In relation to the current study, there are several reasons that may contribute to the lack of difference observed between the treatment groups, including the unexpected rapid onset of action in the comparator group as well as aspects of study design, which highlight the importance of careful design in trials of this type.

Notably, omeprazole with delayed-release characteristics (Losec) improved patient outcomes more rapidly than expected in this study and at the same time, the Zegerid group demonstrated a somewhat slower onset than anticipated. There were, however, various sub-groups of patients, including those based on age, weight and gender, for which an analysis of the primary or secondary endpoints was not carried out. In addition, the ingestion of water during study drug administration and the use of peppermint flavoring could potentially result in an immediate soothing effect that may have impacted the perceived speed of onset of the administered medications, and are further discussed below. Given its delayed-release characteristics, these factors are likely to impact results in the Losec group to a greater extent than for Zegerid suspension, which is designed to act quickly.

Water can provide immediate symptom relief of heartburn; therefore, the ingestion of water during the intake of randomized study medication (suspension and capsule) may have affected the patients’ heartburn severity scores as well as the perceived speed of onset for each medication. One study demonstrated that a glass of water (200mL) increased gastric pH >4 in 10/12 healthy subjects after 1 minute, whereas the median time to pH >4 was 171 minutes for omeprazole[31].

### Limitations

There were a number of limitations to this study. In order to maintain blinding in this double-blind, double-dummy study, the placebo and Zegerid suspensions were flavored with peppermint and peach. It has been reported that ingesting spearmint does not improve or worsen GERD symptoms[32]. However, in contrast to this report, it has been stated that ingestion of mints can cause reflux symptoms, most likely due to relaxation of the lower esophageal sphincter[3]. A study investigating the use of peppermint oil in irritable bowel syndrome reported heartburn as an adverse effect[33]. Thus, the peppermint flavoring in the Zegerid suspension may have had a detrimental effect on the ability of the formulation to relieve heartburn over a sustained period.

Alternatively, the initial cooling effect of the peppermint flavoring[34] in the placebo suspension may have contributed to the observation that Losec improved patient outcomes more rapidly than expected.

The formulation differences of Zegerid and Losec (suspension vs. capsule) may also have influenced the study results. More rapid action may be expected for the Zegerid suspension than the Losec capsule, which delays release of omeprazole due to the time required for the capsule to dissolve. Similarly, the over-encapsulation of Losec might have contributed to further delay omeprazole release. While it is possible that the administration of placebo suspension together with the Losec capsule altered its pharmacokinetic properties, it remains surprising that the capsule acted so rapidly in the current study.

Although recommended as a valid method of symptom assessment[25], rating of heartburn severity by each patient is subjective and therefore may be subject to a placebo effect. However, the use of an active comparator in this study prevented assessment of the extent of any placebo effect of Losec. Symptom assessment at 5-minute intervals during the first 15 minutes post-dose may also have provided greater insight into the immediate effect of Zegerid and Losec on GERD symptoms.

While a crossover design was not employed in the current study, it has been suggested that, due to the substantial inter-individual variability in intra-gastric pH control[15], studies comparing GERD therapies should be of a crossover design in order to measure therapy responses in the same individuals[15]. Crossover studies also minimize the potential influence of genetic polymorphism on individual pH control and PPI metabolism by CYP450 [15]. In the current study, while patients were excluded due to use of medication for the purpose of eradication of *H. pylori*, the *H. pylori* status of the patients was not determined. However, intragastric pH is affected by *H. pylori* colonization of the gastric corpus, and controlling *H. pylori* status has become standard in performing and interpreting clinical trials of intragastric pH control[14].

## Conclusions

This study supports the previously reported difficulty in correlating intragastric pH with clinical effect in the treatment of GERD. Although preliminary PK and pH evidence demonstrated that Zegerid increases intragastric pH more rapidly than Losec, the superiority of Zegerid was not demonstrated for the primary or secondary endpoints. Despite these findings, it should be noted that Zegerid was effective at relieving the symptoms of GERD, it was well tolerated and no safety concerns were raised. This study highlights the significance of a number of technical considerations for GERD studies, including the importance of appropriate study design; careful selection of placebo; control of liquid intake when taking study medication; the use of a crossover design in which each patient acts as their own control; and the assessment of the *H. pylori* status of patients.

## Supporting Information

S1 Protocol (PDF)

S1 CONSORT Checklist (DOC)

S1 File Statistical Analysis Plan. (PDF)
