# Supplementary material for: Challenges of Correlating pH Change with Relief of Clinical Symptoms in Gastro Esophageal Reflux Disease: A Phase III, Randomized Study of Zegerid versus Losec
Source: PLoS One. 2015 Feb 23;10(2):e0116308. doi: 10.1371/journal.pone.0116308 (PMC4338010; doi:10.1371/journal.pone.0116308)
Supplement: S1 Protocol — (PDF) [file pone.0116308.s001.pdf]

NORGINE

Zegerid®
ZEG-01/2010 (GERD)

Protocol

A Phase III, Multi-centre, Double-blind, Double-dummy, Randomised, Study to Assess the Superiority of Zegerid® 20 mg vs. Losec® 20 mg in the Rapid Relief of Heartburn Associated with GERD as on Demand Therapy

Version: 1.0

Version Date: 28 Sep 2010

EudraCT Number: 2010-022082-10

Sponsor:
Norgine Ltd.
Clinical Development
Norgine House, Widewater Place, Moorhall Road
Harefield, Uxbridge, UB9 6NS
United Kingdom

Sponsor Representative:
Dr. Hans-Jürgen Gruss
Tel.: +44 (0) 1895 453 725
Fax: +44 (0) 1895 453 729

Principal Investigator:
Prof. Jarosław Reguła
Department of Gastroenterology and Hepatology
Oncology Centre,
Roentgena Str. 5, 02-781 Warszawa
Poland
Tel.: +44 (0) 48 22 546 23 28
Fax: +44 (0) 48 22 546 30 35

Confidentiality Statement

The information contained within this document is confidential and is the property of Norgine Ltd. This material should only be used in connection with matters authorised by Norgine Ltd. The information should not be disclosed to others without prior written authorisation of Norgine's representative.

Table of Contents

1 APPROVAL SIGNATURES 5
2 ADDRESS LIST 7
3 SYNOPSIS 9
4 LIST OF ABBREVIATIONS 17
5 INTRODUCTION 19
5.1 Background 19
5.2 Rationale 19
5.3 Risk/Benefit Analysis 20
6 STUDY OBJECTIVES 22
7 STUDY DESIGN 24
7.1 Overall Study Design and Plan 24
7.2 Study Sites 25
7.3 Point of Contact 26
8 STUDY POPULATION 27
8.1 Inclusion Criteria 27
8.2 Exclusion Criteria 27
8.3 Patient Drop-outs 29
8.4 Patient Withdrawal 29
8.5 Premature Termination of a Study Site 29
8.6 Premature Termination of the Study 30
8.7 Patient Replacement 30
9 STUDY MEDICATION 31
9.1 Treatments Administered 31
9.2 Identity of Investigational Products 31
9.2.1 Active Study Medication 31
9.2.2 Comparator Study Medication 31
9.2.3 Placebo Medication 32
9.2.4 Antacid Rescue Medication 32
9.3 Labelling and Packaging 32
9.4 Dispensing and Storage 33
9.5 Accountability for the Study Medication 33
9.6 Randomisation 33
9.7 Blinding 34
9.8 Treatment Compliance 34
10 PRIOR AND CONCOMITANT MEDICATION AND OTHER RESTRICTIONS 35
10.1 Prior Medication 35
10.2 Concomitant Medication 35
10.3 Other Restrictions 35
11 CONDUCT OF THE STUDY AND METHODS OF ASSESSMENT 36
11.1 Demographic Data and Medical History 37
11.2 Electronic Diary 38
11.3 Electronic Data Capture 38
11.4 Safety Assessments 38
11.4.1 Adverse Events 38
11.4.2 Clinical Laboratory Tests 38
11.4.3 Physical Examination 39
11.4.4 Vital Signs 40
12 DATA COLLECTION AND ENTRY 41
12.1 Monitoring 41
12.2 Data Management 41

12.2.1 Data Entry 42
12.2.2 Data Coding 42
12.2.3 Data Validation 43
12.2.4 Disclosure of Randomisation Code/Database Lock 43
13 STATISTICAL ANALYSIS 44
13.1 Primary Endpoint 44
13.2 Secondary Endpoints 44
13.3 Definition of Study Populations for Analysis 45
13.3.1 Safety Population 45
13.3.2 Modified Intention-to-Treat Population 45
13.3.3 Per Protocol Population 45
13.4 Determination of Sample Size 45
13.5 Statistical Methods 45
13.5.1 Descriptive Statistics 45
13.5.2 Analysis of Baseline Data 46
13.5.3 Analysis of Efficacy 46
13.5.4 Planned Safety Analyses 47
13.5.5 Interim Analyses 48
14 ADVERSE EVENTS, SERIOUS ADVERSE EVENTS AND REPORTING 49
14.1 Disease Progression 49
14.2 Categorisation of Adverse Events 49
14.2.1 Intensity Classification 49
14.2.2 Causality Classification 49
14.2.3 Treatment-Emergent Adverse Events 50
14.2.4 Assessment of Expectedness 50
14.2.5 Laboratory Test Abnormalities 50
14.2.6 Abnormal Physical Examination Findings 50
14.2.7 Other Investigation Abnormal Findings 51
14.3 Recording and Follow-up of Adverse Events 51
14.4 Serious Adverse Events 51
14.4.1 Definition 51
14.4.2 Reporting Requirements 53
14.4.3 Mandatory Information for Reporting an SAE 53
14.4.4 Reporting Exemptions 54
14.5 Pregnancy 54
14.6 Deaths 54
14.7 Discontinuation/Withdrawal due to Adverse Events/Serious Adverse Events 55
14.8 Reporting to Competent Authorities/IECs/IRBs/Other Investigators 55
14.9 Safety Laboratory 55
15 ETHICAL CONSIDERATIONS AND INSURANCE 56
15.1 Patient Information and Informed Consent 56
15.2 Ethics Committee(s) 56
15.3 Amendments to the Protocol 56
15.4 Finance and Insurance 56
15.5 Regulatory Requirements 57
16 GENERAL OBLIGATIONS, AGREEMENTS AND ORGANISATION 58
16.1 Investigators Brochure 58
16.2 Data Protection and Confidentiality of the Investigator 58
16.3 Electronic CRFs and Handling 58
16.4 Storage of Study Documents and Investigator Site File 59
16.5 Confidentiality 59
16.6 Publication 59
17 QUALITY ASSURANCE/AUDIT 60

18 REFERENCES 61
19 SCHEDULE OF ASSESSMENTS/STUDY FLOWCHART 63
19.1 Schedule of Assessments 63
19.2 Study Flowchart 63
20 APPENDICES 64
20.1 Declaration of Helsinki: 64

1 APPROVAL SIGNATURES

STUDY TITLE: A Multi-centre, Double-blind, Double-dummy, Randomised, Study to Assess the Superiority of Zegerid® 20 mg vs. Losec® 20 mg in the Rapid Relief of Heartburn Associated with GERD as on Demand Therapy

STUDY NUMBER: ZEG-01/2010 (GERD)

EUDRACT NUMBER: 2010-022082-10

I, the undersigned, have read this protocol and confirm that to the best of my knowledge it accurately describes the planned conduct of the study.

SIGNATURE: DATE:

....

Prof. Jarosław Reguła
Principal Investigator

.... 04. Oct. 2010

Dr. Hans-Jürgen Gruss
Sponsor Representative

INVESTIGATOR SIGNATURE PAGE

STUDY TITLE:	A Multi-centre, Double-blind, Double-dummy, Randomised, Study to Assess the Superiority of Zegerid® 20 mg vs. Losec® 20 mg in the Rapid Relief of Heartburn Associated with GERD as on Demand Therapy
STUDY NUMBER:	ZEG-01/2010 (GERD)
EUDRACT NUMBER:	2010-022082-10

The undersigned acknowledges possession of and has read the information on the study medication.

- He/she agrees to use the study material, including medication, only as specified in the protocol. He/she understands that changes cannot be made to the protocol without prior written approval of Norgine Ltd.
- He/she agrees to report to Norgine Ltd. within the specified timeframe any significant clinical Adverse Events (AEs) or abnormal laboratory values that are serious, whether considered related to the administration of study medication or not.
- He/she agrees to comply with regulatory requirements for the monitoring and auditing of this study.

In addition, he/she agrees that the study will be carried out in accordance with the Declaration of Helsinki 2008 and the local laws and regulations relevant to the use of new therapeutic agents.

I, the undersigned, have carefully read this protocol and agree that it contains all the necessary information required to conduct the study.

________________________ ________________________

Signature Date

Investigator's name and address (stamp):

2 ADDRESS LIST

Principal Investigator

Name:	Prof. Jarosław Reguła
Address:	Department of Gastroenterology and Hepatology, Oncology Centre, Roentgena Str. 5, 02-781 Warszawa, Poland
Tel.:	+44 (0) 48 22 546 23 28
E-mail:	jregula@coi.waw.pl

Sponsor Representative

Name:	Dr. Hans-Jürgen Gruss
Address:	Norgine Ltd. Norgine House, Widewater Place, Moorhall Road, Harefield, Uxbridge, UB9 6NS, United Kingdom
Tel.:	+44 (0) 1895 453 725
Fax:	+44 (0) 1895 453 729
E-mail:	hgruss@norgine.com

If the Sponsor's representative cannot be reached, the following dedicated 24-hour emergency number can be used to contact a Norgine trial physician (NTP) for resolution of urgent safety questions +44 (0) 1748 828 787.

Sponsor Study Director

Name:	Dr. Kim Prescott
Address:	Norgine Ltd. Norgine House, Widewater Place, Moorhall Road, Harefield, Uxbridge, UB9 6NS, United Kingdom
Tel:	+44 (0) 1895 453 612
E-mail:	kprescott@norgine.com

Sponsor Project Manager

Name:	Deborah Jones
Address:	Norgine Ltd. Norgine House, Widewater Place, Moorhall Road, Harefield, Uxbridge, UB9 6NS, United Kingdom
Tel.:	+44 (0) 1895 826 636
E-mail:	djones3@norgine.com

CRO Project Manager

Name:	[redacted]
Address:	Premier Research Group Ltd. Rubra 2, Mulberry Business Park, Fishponds Road, Wokingham, RG41 2GY, United Kingdom
Tel.:	+44 (0) 1189 364 019
E-mail:	[redacted]

CRO for Monitoring

Address:	Premier Research Group Ltd. Rubra 2, Mulberry Business Park, Fishponds Road, Wokingham, RG41 2GY, United Kingdom

3 SYNOPSIS

Study Number:	ZEG-01/2010 (GERD)
Study Drug(s):	ZEGERID® 20 mg powder for suspension/Losec® 20 mg capsule
Title:	A double-blind, double-dummy, randomised, study to assess the superiority of Zegerid® 20 mg vs. Losec® 20 mg in the rapid relief of heartburn associated with GERD as on demand therapy.
Phase:	Phase III Study
EudraCT Number:	2010-022082-10
Study Population:	Approximately 300 patients with a history of GERD who have completed PPI therapy and are previously symptomatically responsive to PPI therapy with standard dose and continue to experience heartburn 2-3 days per week, will be enrolled to obtain 194 evaluable patients.
Study Design:	This is a multi-centre, double-blind, double-dummy, randomised study in patients with heartburn associated with GERD. Patients will be randomised to receive one of the following treatments:<br>• 20 mg Zegerid® suspension plus over-encapsulated placebo capsule or<br>• 20 mg Losec® capsule over-encapsulated plus placebo suspension to assess the superiority of Zegerid® versus Losec® in the rapid relief of heartburn.<br>Patients will also receive Gaviscon® (rescue medication), which will be taken as required for up to 2 doses per day (except for the first 180 minutes of each of the 3 episodes of heartburn, which are being recorded by e-diary).<br>The study will be performed in approximately 50 study sites in 6 EU countries.
Study Plan:	The duration of the study for each patient will be a maximum of 21 days:<br>Beginning with Screening Visit (Visit 1), patients will start a 1 week baseline symptom assessment period, during which patients will record daily episodes of heartburn using an electronic diary (e-diary) and a 9-point Likert severity scale for 7 days. During this time, patients will be requested not to take antacid medication except Gaviscon® (rescue medication) for up to 2 doses per day, which will be provided by the Investigator.<br>Patients will receive full instructions and training for use of e-diaries to enable them to record heartburn severity scores and usage of Gaviscon® (rescue medication). Follow-up assistance will be available through e-diary call centres. The e-diary service provider will issue user and trainer

	instruction manuals to each site. Staff members involved with the study will provide training to patients at Visit 1 (Screening Visit). Blood samples will also be taken at Visit 1.<br><br>Patients will return for Visit 2, 7 days post the Screening Visit when eligibility will be checked and they will be randomly assigned to one of the two treatment arms. This will be followed by a maximum 14 day period, during which eligible patients will receive one of the two randomly assigned treatments. Patients will be provided with a maximum of 3 days of randomised study medication and will be instructed to take 1 dose of randomised study medication immediately when they experience an episode of heartburn (maximum 1 dose per day for a total of 3 days out of 14 days).<br><br>For every episode of heartburn (between 06:00 and 22:00 hours), randomised study medication provided should be taken. Patients will be required to record the severity of the heartburn episode and to record the time and date (pre-dose) using an e-diary as provided (patients should take randomised study medication for episodes of heartburn occurring between 06:00 and 22:00 hours (or until they go to bed), but should continue recording for the 180 minutes after taking randomised study medication). Patients will be requested to grade the severity of the heartburn at 15, 30, 45, 60, 75, 90, 105, 120, 150 and 180 minutes after taking the randomised study medication (severity scores will be recorded for a total of 3 hours) using their e-diary.<br><br>Patients should only take 1 dose of randomised study medication when they experience an episode of heartburn. If the patient experiences more than 1 episode in a day then they should use Gaviscon® (rescue medication) as provided for up to a maximum of 2 doses per day, but not within the initial 3 hours after dosing with randomised study medication.<br><br>Patients will also be required to keep a record of the Gaviscon® (rescue medication) usage during the 14 day randomised treatment period. Gaviscon® (rescue medication) should not be used for the first 180 minutes (3 hours) of each episode of heartburn being recorded by e-diary, where randomised study medication has been taken.<br><br>After 3 episodes of heartburn on 3 separate days where randomised study medication has been taken and recorded on e-diaries, patients should contact the Principal Investigator. Patients will participate for a maximum of 14 days post randomisation to ensure 3 days of heartburn treatment are recorded, however patient participation may end prematurely if 3 episodes of heartburn on 3 separate days are recorded before the 14th day.<br><br>Patient reported outcomes will be recorded using the e-diary by the patients when they have an episode of heartburn and have taken randomised study medication.<br><br>1. Severity of heartburn at time zero just prior to randomised study medication being taken.<br>2. The severity of heartburn recorded at 15, 30, 45, 60, 75, 90, 105, 120, 150 and 180 minutes after the start of randomised study medication for each episode.<br>3. The concomitant use of Gaviscon® (rescue medication) during the 14

	day randomised treatment period.<br><br>Patients will return for Visit 3 on Day 15 (± 3 days) after the Randomisation Visit where the e-diary, adverse events and concomitant medications will be reviewed.
Objectives:	The objective of this study is to compare Zegerid® 20 mg suspension to Losec® 20 mg capsule with respect to the following:<br><br>Primary:<br>Determine the median time to sustained response, which is defined as a reduction of severity of heartburn associated with gastroesophageal reflux disease (GERD) by 3 points or more on a 9-point Likert severity scale, which is sustained for 45 minutes or more.<br>Key secondary:<br>1. Determine the median time to sustained partial response, defined as a reduction of 2 points or more on the 9-point Likert severity scale, which is sustained for 45 minutes or more.<br>2. Determine the median time to sustained total relief, defined as zero severity (no heartburn) on the 9-point Likert severity scale, which is sustained for 45 minutes or more.<br>3. Determine the proportion of patients who have achieved sustained response, sustained partial response or sustained total relief by 45, 60 and 90 minutes.<br>Secondary:<br>1. Determine the severity of heartburn associated with GERD and change in severity from pre-dose at all time points.<br>2. Determine the proportion of patients who have achieved sustained response, sustained partial response or sustained total relief at all other time points.<br>3. Determine the proportion of patients with total relief (defined as zero severity on the 9-point Likert severity scale) of heartburn associated with GERD at all time points.<br>4. Determine the proportion of patients with response (defined as a change in the severity of heartburn from pre-dose of 3 or more points on the 9-point Likert severity scale) at all time points.<br>5. Determine the proportion of patients with at least partial response (defined as a change in the severity of heartburn from pre-dose of 2 or more points on the 9-point Likert severity scale) at all time points.<br>6. The area under the severity-time curve (AUC) will be calculated for the for the following time periods; 0-60, 0-120 and 0-180 minutes<br>7. Determine the usage of Gaviscon® (rescue medication) over the 14 day randomised treatment period.<br>8. Safety and tolerability.
Inclusion Criteria:	Study patients will be included in the study if they satisfy the following criteria:<br><br>1. Male or female, between 18 and 75 years old.<br>2. History of frequent episodes of heartburn associated with GERD for at least 2-3 days per week during 2-4 weeks before screening and have responded to standard PPI therapy in the past 12 months.<br>3. Have not taken on-demand PPI therapy for >3 consecutive days

	within 4 weeks before the screening period.<br>4. The patient's written informed consent must be obtained prior to inclusion.<br>5. Willing and able to complete the entire procedure and to comply with study instructions.<br>6. Females of childbearing potential must employ an adequate method of birth control.<br><br>Inclusion criteria applicable to Screening period:<br>1. Recorded at least 1 evaluable episode of heartburn on 2 separate days at level 4 or higher on the Likert severity scale prior to randomisation.<br>2. Competence in the use and completion of e-diary documentation.
Exclusion Criteria:	Study patients will be excluded if they meet any of the following criteria:<br>1. Age <18 or >75 years old.<br>2. Intake of any medication for the purpose of the eradication of *H. pylori* during the last 28 days before the start of the study.<br>3. Intake of systemic glucocorticoids or non-steroidal anti-inflammatory drugs (NSAIDs) including COX-2-inhibitors (≥3 consecutive days per week) during the last 28 days before the start of the study; except regular intake of enteric coated aspirin dosages up to 150 mg/d.<br>4. Previously underwent acid-lowering surgery or other surgery of the oesophagus and/or upper gastrointestinal tract (excluding: appendectomy, cholecystectomy and polypectomy).<br>5. History of co-existing disease that affects the oesophagus (e.g. Barrett's oesophagus, Zollinger-Ellison syndrome, oesophageal stricture), and have undergone an endoscopy with results of incomplete healing of erosions following standard PPI therapy within the last 3 months.<br>6. History of active gastric or duodenal ulcers within 3 months of the first dose of the study drug or had acute upper gastrointestinal (GI) bleeding within last 6 months.<br>7. Documented presence of severe renal or hepatic insufficiency.<br>8. Known hypersensitivity to omeprazole.<br>9. Concurrent participation in a study with an investigational drug or participation within 30 days of study entry.<br>10. Females who are pregnant, or planning a pregnancy. Females of child bearing potential not using reliable methods of birth control (the use of the combined oral pill, progestogen only pill, combined contraceptive patches, depot contraceptives or double barrier method all are acceptable as a reliable form of contraception).<br>11. Clinically significant laboratory abnormality or disease which, in the opinion of the Investigator, will create a risk for the patient, obscure the effects of study treatment or interfere with study results.<br>12. Received any of the following drugs within 2 weeks before the first dose of randomised study medication or needed these drugs for continuous concurrent therapy: theophylline, bismuth salts, warfarin, phenytoin, barbiturates, antineoplastic agents, erythromycin, clarithromycin, sucralfate or nelfinavir.<br>13. Taking concomitant medications that rely on the presence of gastric acid for optimal absorption (e.g. ketoconazole).

	14. Onset of psychiatric medication (e.g. depressants, stimulants or hallucinogens) in the previous 6 months and during the entire course of the study.<br><br>Exclusion criteria applicable to Screening period:<br>1. Recorded <1 episode of heartburn on 2 separate days at level 4 or higher on the Likert severity scale prior to randomisation.<br>2. Completing <90% (9 out of 10) of the time points with evaluable data on their e-diary.
Treatment Schedule:	• Patients should only take 1 dose of randomised study medication each day when they experience an episode of heartburn for a maximum of 3 days over the 14 day randomised treatment period.<br><br>• The patient will be provided with a maximum of 3 doses of randomised study medication for use during the 14 day randomised treatment period.<br><br>• If the patient experiences more than 1 episode of heartburn in a day when they have taken randomised study medication, then they can use Gaviscon® (rescue medication) after the 3 hour follow-up period (Gaviscon® [rescue medication] will be provided). Gaviscon® (rescue medication) can not be used for the first 180 minutes of each episode of heartburn being recorded by e-diary.<br><br>• If the patient experiences symptoms of heartburn outside of the 3 days of taking randomised study medication, they can use study allocated Gaviscon® (rescue medication) for up to 2 doses per day.<br><br>• Each of the 3 episodes of heartburn where randomised study medication is taken should be recorded using the e-diary provided.
Safety Parameters:	• Adverse Events<br><br>• Laboratory Safety Tests: Blood samples will be taken at Visit 1 (Screening Visit) to confirm patient eligibility for the study<br><br>• All blood samples for the safety laboratory tests will be evaluated at the local study site laboratory. Sample collection will be carried out according to each local laboratory practice. Each blood sample will be 20 mL or less in volume. Any blood results falling outside of the laboratory normal range will be flagged and reviewed by the Investigator. If there are any abnormalities that the Investigator considers to be clinically significant, the patient should not be included in the study. If additional laboratory tests are carried out during the study that, in the Investigator's opinion, show clinically significant or pathological changes during or after termination of the treatment, these should be reported as AEs and have to be followed with appropriate medical care, even after termination of the study, until normal or baseline values are reached and the condition has stabilised or a non-study medication cause has been identified. The following parameters will be determined as a minimum:

	• Biochemistry: Sodium, Potassium, Urea, Creatinine, Bicarbonate, Chloride, Alanine Transaminase (ALT), Asparate Aminotransferase (AST), Alkaline Phosphate (ALP), Albumin, Total Protein, Bilirubin Haematology: Haemoglobin, Haematocrit, Erythrocytes, (RBC), Mean Cell Haemoglobin (MCH), Leukocytes (WBC), Differential Count, Platelet.<br><br>• Urine samples will be collected from women of child-bearing potential and a urine pregnancy test will be carried out at Visit 1 (Screening Visit). Any woman with a positive pregnancy test result must be withdrawn from the study. The following parameters will be determined as a minimum: Urinalysis: Glucose, Protein, Creatinine, Sodium, Potassium, Bilirubin, Urine Specific Gravity, Erythrocytes, Leukocytes.<br><br>• Physical Examination: A full physical examination will be performed at Visit 1 (Screening Visit), with any changes from this assessment then being noted in the e-CRF at the subsequent study visits (Visits 2 and 3). The following organ systems will be examined: cardiovascular, respiratory, abdominal, genitourinary, gastrointestinal, musculoskeletal, neurological and dermatological.<br><br>• Vital Signs: Blood pressure (BP: Systolic [SBP]/Diastolic [DBP]) and pulse rate (PR) will be measured at the following visits: Visit 1 (Screening Visit) and Visit 2 (Randomisation Visit). Body temperature will be documented at the Visit 1 and Visit 2: temperature will be recorded in degree Celsius (°C). Time and date of temperature measurement will be fully documented.
Statistical Analysis:	Safety Population: The Safety population will consist of all randomised patients who received at least one dose of randomised study medication.<br><br>Modified Intention-to-Treat Population: The Modified Intention-to-Treat (mITT) population will consist of all patients in the Safety population who are evaluable (recorded data for at least one evaluable episode of heartburn).<br><br>Per Protocol Population: The Per Protocol (PP) population will consist of all patients included in the mITT population but will exclude the following:<br>• Patients violating any inclusion/exclusion criterion<br>• Patients with major protocol violations (e.g., poor compliance, incorrect completion of e-diaries) in a sufficiently serious manner to warrant exclusion.<br><br>Statistical Methods:<br><br>Descriptive Statistics: For dichotomous and categorical variables, absolute and relative frequencies (counts and percents) will be calculated. For continuous variables, comprehensive data summaries will be presented with sample characteristics (n, arithmetic mean, standard deviation [SD], minimum, lower quartile, median, upper quartile and maximum) for all continuous variables by treatment. Where data are collected over time, both the observed data and the change from the run-in period will be summarised

	at each time point.<br><br>Analysis of Baseline Data: Baseline characteristics will be summarised by treatment for each population. There will be no statistical testing of the comparability of the populations.<br><br>Analysis of Efficacy: The primary efficacy hypothesis will be evaluated in a confirmatory test.<br><br>Each patient will have data from between 1 and 3 episodes of heartburn that have been treated with the study medication. The median will be used to give an average severity of heartburn for each patient at each time point.<br><br>An evaluable episode will be one with a baseline severity of level 4 or more and no more than 1 missing time point in the 3 hour period. If rescue medication is taken in the 3 hour period, the baseline observation carried forward method will be used imputing the baseline severity at all time points following the first intake of rescue medication and the episode will be considered evaluable. The patient will be considered a non-responder at all later time points.<br><br>A patient will be evaluable if they have at least one evaluable episode<br><br>All efficacy analyses will use the mITT population and the PP population.<br><br>Primary Efficacy Analysis - The primary efficacy parameter is the time to sustained response, which is defined a reduction of 3 or more points on the 9-point Likert severity scale which is sustained for 45 minutes or more. This will be defined using the median heartburn severity at each time point. Patients who do not achieve sustained response will be censored at the last evaluable time point. The time to the start of this response will be shown in each treatment group using a Kaplan-Meier curve. The median time and the 95% confidence interval will be presented for each treatment. Differences between the two treatments will be tested at a two-sided significance level of 0.05 using a Cox regression model, including a centre effect. A 95% confidence interval will be presented for the treatment hazards ratio.<br><br>Secondary Efficacy Analysis (key secondary efficacy parameters) - The time to sustained partial response and the time to sustained total relief will be analysed in the same way as the primary efficacy parameter.<br><br>The proportion of patients who have achieved sustained response, sustained partial response or sustained total relief in each treatment group by 45, 60 and 90 minutes will be presented from the Kaplan-Meier analysis. A 95% confidence interval will be presented for the treatment difference at each time point.<br><br>Secondary Efficacy Analysis (secondary efficacy parameters) - The severity of heartburn and change from baseline will be summarised with descriptive statistics for each treatment at each time point.<br><br>The AUCs will be summarised with descriptive statistics for each treatment at each time point.<br><br>The proportion of patients who have achieved sustained response, sustained partial response or sustained total relief in each treatment group at all other

	time points will be presented from the Kaplan-Meier analysis.<br><br>The proportion of patients who have achieved response, partial response or total relief in each treatment group at all time points will be summarised by treatment in frequency tables.<br><br>The total use of Gaviscon® (rescue medication) over the 14 day randomised treatment period will be summarised as the number of doses taken. The proportion of days where Gaviscon® (rescue medication) was taken and the average number of doses taken per day will be presented.<br><br>No testing is planned for any of these secondary parameters, although further exploratory analyses may be added. These will be described in the Statistical Analysis Plan.<br><br>Planned Safety Analyses: All safety analyses will use the Safety population.<br><br>The primary safety parameter is the occurrence of SAEs and AEs. All data will be summarised within each treatment group. All SAEs and AEs will be listed using coding for System Organ Class and Preferred Term (using the MedDRA version 13.0 or higher). Only treatment-emergent AEs will be included in the analysis.<br><br>SAEs and AEs will be summarised as follows:<br><br>• Number and percentage of patients with AEs classified by System Organ Class and Preferred Term<br>• Number and percentage of patients with AEs by strongest relationship to randomised study medication, System Organ Class and Preferred Term<br>• Number and percentage of patients with AEs by maximum severity, System Organ Class and Preferred Term<br>• Number and percentage of patients with serious adverse events (SAEs) classified by System Organ Class and Preferred Term<br>• Number and percentage of patients with drug-related SAEs classified by System Organ Class and Preferred Term.<br><br>Interim Analyses: No interim analysis is planned for this study.

4 LIST OF ABBREVIATIONS

ATPase	Adenosine Triphosphatase
AE	Adverse Event
ALP	Alkaline Phosphate
ALT	Alanine Transaminase
AST	Asparate Aminotransferase
AUC	Area Under the Curve
BP	Blood Pressure
CA	Competent Authorities
CAP	Community-Acquired Pneumonia
CRO	Contract Research Organisation
CYP2C19	Cytochrome P450 2C19
e-CRF	Electronic Case Report Form
EDC	Electronic Data Capture
EU	European Union
GCP	Good Clinical Practice
GERD	Gastroesophageal Reflux Disease
GI	Gastrointestinal
H_2RA	H_2 Receptor Antagonist
ICH	International Conference on Harmonisation
IEC	Independent Ethics Committee
IMPD	Investigational Medicinal Product Dossier

IRB	Institutional Review Board
IWRS	Interactive Web Response System
MCH	Mean Cell Haemoglobin
MedDRA	Medical Dictionary for Regulatory Activities
mITT	Modified Intention-to-Treat
NSAIDs	Non-Steroidal Anti-Inflammatory Drugs
NTP	Norgine Trial Physician
OTC	Over-The-Counter
PP	Per Protocol
PPI	Proton Pump Inhibitor
PR	Pulse Rate
QC	Quality Control
RBC	Red Blood Cell
SAE	Serious Adverse Event
SDV	Source Data Verification
SmPCs	Summary of Product Characteristics
SUSARs	Suspected Unexpected Serious Adverse Reactions
TMF	Trial Master File
WBC	White Blood Cell
WHO	World Heath Organisation

5 INTRODUCTION

5.1 Background

Omeprazole is acid-labile and is rapidly degraded by gastric acid. Like other marketed oral proton pump inhibitors (PPIs), omeprazole is delivered with an enteric coating to protect it from degradation upon exposure to acid. Because of their enteric coatings, these formulations have delayed-release characteristics.

Omeprazole belongs to the class of antisecretory compounds, substituted benzimidazoles that suppress production of gastric acid, but do not exhibit anticholinergic or histamine-2 receptor antagonist (H_2RA) properties. They suppress gastric acid secretion by specific inhibition of the H+/K+ adenosine triphosphatase (ATPase) enzyme system at the secretory surface of the gastric parietal cell. This effect is dose related and leads to inhibition of both basal and stimulated acid secretion irrespective of the stimulus.

Zegerid® is a novel fixed combination of omeprazole and antacid sodium bicarbonate, with immediate-release characteristics. In this product, the sodium bicarbonate neutralises gastric acid and, protects the omeprazole from gastric acid degradation, so that the PPI can be rapidly absorbed. The immediate-release formulation has been developed to be administered as oral suspension or in solid dosage forms (oral capsule).

Previous studies were conducted to identify pharmacodynamic characteristics that demonstrate a difference for Zegerid® formulations from delayed release PPIs, such as Losec® (Prilosec®). Zegerid® does not need to be dosed prior to a meal to provide effective control of gastric acidity. A number of studies were conducted to characterize the night-time pH profile after bedtime dosing, and to compare the effects of bedtime dosing with Zegerid® and delayed release PPIs on the control of nocturnal gastric acidity. These studies have shown that the night-time pH profile after bedtime dosing with Zegerid® is different from the night-time pH profile after dosing with delayed-release PPIs. Further studies were conducted to show the effects of morning dosing with immediate-release PPIs and delayed-release PPIs in 24-hour control of gastric acidity. Morning dosing with Zegerid® provides significantly better 24-hour gastric acid control when compared to delayed-release PPIs (Lansoporazole and Pantoprazole).[1]

5.2 Rationale

Heartburn (also referred to as pyrosis or acid indigestion) is the hallmark symptom for GERD and, accompanied with acid regurgitation and other symptoms has a substantial negative impact on a patients' quality of life.[2] A recent publication has shown patients who respond best to therapy subsequently enjoy the best quality of life.[3]

Although various medications are currently available (including OTC antacids, H_2RAs and PPIs), studies show there is still a need for more effective therapy to fill the gap that exists between patients' expectations and their experience.[4] Failure of PPIs to completely resolve symptoms has become a commonly encountered clinical problem in gastroenterology practices. In a previous study, approximately 25% of the patients with GERD continued to have heartburn symptoms despite treatment with standard dose PPI once daily.[5]

Patients who continue to report bothersome GERD symptoms whilst receiving once-daily PPI therapy will most commonly have their PPI dosage doubled, be given omeprazole twice daily, or have their dosing period extended. This clinical approach has become the standard of care, and it is based on the assumption that most patients with refractory symptoms while receiving PPIs still have some level of abnormal distal oesophageal acid exposure.[3]

5.3 Risk/Benefit Analysis

An occasional bout of heartburn (or indigestion) is generally not clinically significant; however there are concerns about the long-term effects of untreated, frequent or troublesome heartburn; including sleep disturbance, chronic cough and even asthma.[6] Chronic heartburn associated with GERD can cause irreversible structural changes to the lining of the oesophagus (Barrett's Oesophagus), putting patient's at a higher risk of developing oesophageal adenocarcinoma (oesophageal cancer).

Further studies concerning the use of PPI have been conducted investigating the association between GERD and due the development of gastrointestinal neoplasia and malabsorption of nutrients.[7] The use of PPI had previously been associated with an increased risk of infections in the lower gastrointestinal tract (due to *Salmonella*, *Clostridium difficile*);[8] and recent studies have been conducted to assess the association between PPI use and community-acquired pneumonia (CAP). The outcomes of these studies suggest that short-term use of PPIs (within last 30 days) increased the risk of CAP; however longer-term use of PPI did not increase the risk of CAP.[9]

Omeprazole has a well established safety profile. The fixed dose combination of omeprazole and sodium bicarbonate in Zegerid® has been demonstrated to be effective in reducing the amount of acid production in the stomach. The most frequently reported adverse events associated with Zegerid® therapy during controlled clinical studies were headache, diarrhoea and abdominal pain, which were mild or moderate in severity and reversible on cessation of treatment.

Long-term use of omeprazole comes with its own risks. Recent studies indicate that patients on long-term omeprazole experience a significant increase in serum gastrin levels. Hyperplasia of enterochromaffin-like cells with no evidence of dysplasia, carcinoid tumours, or other neoplastic changes, has been observed in patients after

5 years of treatment. Long term treatment with omeprazole is not considered a risk for gastric carcinogenesis.[10] Other studies have confirmed the association between long-term use of PPIs and an increased risk of osteoporosis. Duration of PPI exposure exceeding 5 years is associated with an increased risk of hip fracture and exposure exceeding 7 years is associated with a significantly increased risk of osteoporosis-related fractures.[11]

This study requires only 3 days of treatment with a single daily dose of omeprazole or omeprazole and sodium bicarbonate. While this will have no long-term benefit for the patients with heartburn, the risks associated with this amount of the study drug are negligible.

Based on this information, the benefits of treatment and the potential avoidance of further complications outweigh the possible risks associated with short-term use of Zegerid®.

The study will be conducted according to the ICH-GCP (International Conference on Harmonisation - Good Clinical Practice) guidelines and EU Clinical Trials Directive. Prior to the start of the study, Ethics Committee and competent authority approvals will be obtained.

6 STUDY OBJECTIVES

The objective of this study is to compare Zegerid® 20 mg suspension to Losec® 20 mg capsules with respect to the following:

Primary:

Determine the median time to sustained response, which is defined as a reduction of severity of heartburn associated with gastroesophageal reflux disease (GERD) by 3 points or more on a 9-point Likert severity scale, which is sustained for 45 minutes or more.

Key secondary:

1. Determine the median time to sustained partial response, defined as a reduction of 2 points or more on the 9-point Likert severity scale, which is sustained for 45 minutes or more.
2. Determine the median time to sustained total relief, defined as zero severity (no heartburn) on the 9-point Likert severity scale, which is sustained for 45 minutes or more.
3. Determine the median proportion of patients who have achieved sustained response, sustained partial response or sustained total relief by 45, 60 and 90 minutes.

Secondary:

1. Determine the severity of heartburn associated with GERD and change in severity from pre-dose at all time points.
2. Determine the proportion of patients who have achieved sustained response, sustained partial response or sustained total relief at all other time points.
3. Determine the proportion of patients with total relief (defined as zero severity 9-point Likert severity scale) of heartburn associated with GERD at all time points.
4. Determine the proportion of patients with response (defined as a reduction in the severity of heartburn from pre-dose of 3 or more points on the 9-point Likert severity scale) at all time points.
5. Determine the proportion of patients with at least partial response (defined as a reduction in the severity of heartburn from pre-dose of 2 or more points on the 9-point Likert severity scale) at all time points.
6. Determine the area under the severity-time curve (AUC) will be calculated for the for the following time periods; 0-60, 0-120 and 0-180 minutes.

7. Determine the usage of Gaviscon® (rescue medication) over the 14 day randomised treatment period.
8. Safety and tolerability.

7 STUDY DESIGN

7.1 Overall Study Design and Plan

This is a multi-centre, double-blind, double-dummy, randomised study in patients with heartburn associated with GERD. Patients will be randomised to receive one of the following treatment arms:

- 20 mg Zegerid® suspension plus over-encapsulated placebo capsule or
- 20 mg Losec® capsule over-encapsulated plus placebo suspension to assess the superiority of Zegerid® versus Losec® in the rapid relief of heartburn.

Patients will also receive Gaviscon® (rescue medication), which will be taken as required for up to 2 doses per day (except for the first 180 minutes of each of the 3 episodes of heartburn, where randomised study medication has been taken and which are being recorded by using an electronic diary [e-diary]).

The study will be performed in approximately 50 study sites in 6 EU countries.

The duration of the study for each patient will be a maximum of 21 days.

Beginning with baseline Screening Visit (Visit 1), patients will start a 1 week baseline symptom assessment period, during which patients will record daily episodes of heartburn using an e-diary and the Likert severity scale for 7 days. During this time, patients will be requested not to take antacid medication except Gaviscon® (rescue medication), which will be provided by the Investigator (maximum of 2 doses per day).

Patients will receive full instructions and training for use of e-diaries to enable them to record heartburn severity scores and usage of Gaviscon® (rescue medication). Follow-up assistance will be available through e-diary call centres. The e-diary service provider will issue user and trainer instruction manuals to each site. Staff members involved with the study will provide training to patients at Visit 1 (Screening Visit). Blood samples will also be taken at Visit 1.

Patients will return for Visit 2, 7 days post the Screening Visit when eligibility will be checked and they will be randomly assigned to one of the two treatment arms. This will be followed by a maximum 14 day period, during which eligible patients will receive one of the randomly assigned treatments. Patients will be provided with a maximum of 3 days of randomised study medication and will be instructed to take 1 dose of randomised study medication immediately when they experience an episode of heartburn (maximum 1 dose per day for a total of 3 days out of 14 days).

For every episode of heartburn (between 06:00 and 22:00 hours), randomised study

medication provided should be taken. Patients will be required to record the severity of the heartburn episode and to record the time and date (pre-dose) using an e-diary as provided[1]. Patients will be requested to grade the severity of the heartburn at 15, 30, 45, 60, 75, 90, 105, 120, 150 and 180 minutes after taking the randomised study medication (severity scores will be recorded for a total of 3 hours) using their e-diary.

Patients should only take 1 dose of randomised study medication when they experience an episode of heartburn. If the patient experiences more than 1 episode in a day then they should use Gaviscon® (rescue medication) as provided for up to a maximum of 2 doses per day, but not within the initial 3 hours after dosing with randomised study medication.

Patients will also be required to keep a record of the Gaviscon® (rescue medication) usage during the 14 day randomised treatment period. Gaviscon® (rescue medication) will not be used for the first 180 minutes (3 hours) of each episode of heartburn being recorded by e-diary, where randomised study medication has been taken.

After 3 episodes of heartburn on 3 separate days where randomised study medication has been taken and recorded on e-diaries, patients should contact the Principal Investigator. Patients will participate for a maximum of 14 days post randomisation to ensure 3 days of heartburn treatment are recorded, however patient participation may end prematurely if 3 episodes of heartburn on 3 separate days are recorded before the 14th day.

Patient reported outcomes will be recorded by patients using the e-diary, when they have an episode of heartburn and have taken the randomised study medication.

1. Severity of heartburn at time zero just prior to randomised study medication being taken
2. The severity of heartburn recorded at 15, 30, 45, 60, 75, 90, 105, 120, 150 and
3. The concomitant use of Gaviscon® (rescue medication) during the 14 day randomised treatment period.

Patients will return for Visit 3 on Day 15 after the Randomisation Visit where the e-diary, adverse events and concomitant medications will be reviewed.

7.2 Study Sites

It is anticipated that the study will be carried out at a maximum of up to 50 study sites in 6 EU countries. The target enrolment is approximately 300 randomised patients in order to achieve 194 evaluable patients.

[1] Patients should take randomised study medication for episodes of heartburn occurring between 06:00 and 22:00 hours (or until they go to bed), but should continue recording for the 180 minutes after taking randomised study medication

7.3 Point of Contact

The Principal Investigator for this study is Prof. Jarosław Reguła.

For all patients a local point of contact will be provided where they can obtain information on the study, their rights and whom to contact in case of study-related injury. This information will be provided in the Patient Information Sheet and Informed Consent Form.

8 STUDY POPULATION

Approximately 300 patients with a history of GERD who have completed PPI therapy and are previously symptomatically responsive[2] to PPI therapy standard dose and continue to experience heartburn 2-3 days per week, will be enrolled to obtain 194 evaluable patients.

8.1 Inclusion Criteria

Study patients will be included in the study if they satisfy the following criteria:

1. Male or female, between 18 and 75 years old.
2. History of frequent episodes of heartburn associated with GERD for at least 2-3 days per week during 2-4 weeks before screening and have responded to standard PPI therapy in the past 12 months.
3. Have not taken on-demand PPI therapy for >3 consecutive days within 4 weeks before the screening period.
4. The patient's written informed consent must be obtained prior to inclusion.
5. Willing and able to complete the entire procedure and to comply with study instructions.
6. Females of childbearing potential must employ an adequate method of birth control.

Inclusion criteria applicable to Screening period:

1. Recorded at least 1 evaluable episode of heartburn on 2 separate days at level 4 or higher on the Likert severity scale prior to randomisation.
2. Competent in the use and completion of e-diary.

8.2 Exclusion Criteria

Study patients will be excluded if they meet any of the following criteria:

1. Age <18 or >75 years old.
2. Intake of any medication for the purpose of the eradication of Helicobacter pylori (*H. pylori*) during the last 28 days before the start of the study.
3. Intake of systemic glucocorticoids or non-steroidal anti-inflammatory drugs (NSAIDs) including COX-2-inhibitors (≥3 consecutive days per week) during the last 28 days before the start of the study; except regular intake of enteric

[2] Patients with an improvement of symptom on PPI

coated aspirin dosages up to 150 mg/d.

4. Previously underwent acid-lowering surgery or other surgery of the oesophagus and/or upper gastrointestinal tract (excluding: appendectomy, cholecystectomy and polypectomy).
5. History of co-existing disease that affects the oesophagus (e.g. Barrett's oesophagus, Zollinger-Ellison syndrome, oesophageal stricture), and have undergone an endoscopy with results of incomplete healing of erosions following standard PPI therapy within the last 3 months.
6. History of active gastric or duodenal ulcers within 3 months of the first dose of the study drug or had acute upper gastrointestinal (GI) bleeding within last 6 months.
7. Documented presence of severe renal or hepatic insufficiency.
8. Known hypersensitivity to omeprazole.
9. Concurrent participation in a study with an investigational drug or participation within 30 days of study entry.
10. Females who are pregnant, or planning a pregnancy. Females of child bearing potential not using reliable methods of birth control.[3]
11. Clinically significant laboratory abnormality or disease which, in the opinion of the Investigator, will create a risk for the patient, obscure the effects of study treatment or interfere with study results.
12. Received or require any of the following drugs within 2 weeks before the first dose of study or needed these drugs for continuous concurrent therapy: theophylline, bismuth salts, warfarin, phenytoin, barbiturates, antineoplastic agents, erythromycin, clarithromycin, sucralfate or nelfinavir.
13. Taking concomitant medications that rely on the presence of gastric acid for optimal absorption (e.g. ketoconazole).
14. Onset of psychoactive medication (e.g. depressants, stimulants or hallucinogens) in the previous 6 months and during the entire course of the study.

Exclusion criteria applicable to Screening period:

1. Recorded <1 episode of heartburn on 2 separate days at level 4 on the 9-point Likert severity scale during the 7 day screening period prior to randomisation
2. Completing <90% (<9 out of 10) of the time points with evaluable data on the e-diary.

[3] The use of the combined oral pill, progestogen only pill, combined contraceptive patches, depot contraceptives or double barrier method all are acceptable as a reliable form of contraception

8.3 Patient Drop-outs

Drop-outs are defined as those patients who have been enrolled in the study but withdraw or are withdrawn from the study prior to randomisation to taking any randomised study medication. For instance, a patient could drop out if, in the opinion of the Investigator, haematological or biochemical findings from specimens drawn during the run-in period are clinically significant or any other conditions occur that do not allow the patient to participate further into the treatment period of the study. Drop-out patients will be treated according to local standard practice.

8.4 Patient Withdrawal

The patient has the right to abstain from participation in the study or to withdraw consent to participate at any time during the course of the study. Norgine Ltd or the Investigator also holds the responsibility to withdraw a randomised patient from the study prematurely if it is considered in the best interest of the patient. The entire study might be stopped if there are unpredicted safety concerns.

The patient's participation may be discontinued at any point during the study without implications on further medical care if:

- They no longer wish to participate
- The Investigator feels it is in their best interest to withdraw or
- If the patient is not willing to comply with the requirements of the study protocol.

If the participation of any patient ceases prematurely, the reasons leading to withdrawal from the study should be described in detail. Irrespective of the reason for withdrawing, a final medical examination should be performed and documented in the 'End of Study' Visit. The timing of the End of Study Visit for a withdrawn patient should be at the patient's convenience but within two weeks of the last study drug administration. The assessments conducted should be the same as the protocol specified End of Study Visit. An Electronic Case Report Form (e-CRF) must be completed as fully as possible giving reasons for withdrawal when available.

8.5 Premature Termination of a Study Site

A study site may be discontinued by Norgine Ltd. for significant deviations from the protocol or due to difficulties experienced in running the study at that site.

Norgine Ltd. may terminate this study in one particular site or several study sites for one of the following reasons:

- Non-compliance with GCP and/or regulatory requirements
- Site cannot recruit an adequate number of patients
- False records in the e-CRF due to negligence
- Inadequate co-operation with Norgine Ltd., or its representatives
- The Investigator requests closure of his/her study site.

If the study is prematurely terminated in one or more study sites, the relevant Investigators have to inform their patients and take care of appropriate follow-up and further treatment of the patients according to local standard practice. Independent Ethics Committees (IECs) and Regulatory Authorities will be informed about the reasons and time of termination according to applicable laws and regulations.

8.6 Premature Termination of the Study

The study will be terminated prematurely if:

- New toxicological or pharmacological findings, serious AEs or frequent AEs that invalidate the positive benefit-risk assessment
- AEs occur in such prominence (i.e. severity and frequency) that the proposed schedule can no longer be adhered to
- Rate of recruitment is inadequate to ascertain a timely and orderly completion of the study within a reasonable time frame
- Significant protocol deviations occur at a frequency implicating the valid and safe conduct of the study
- Norgine Ltd. decides to discontinue the study.

8.7 Patient Replacement

Patients who are discontinued prematurely will not be replaced.

9 STUDY MEDICATION

9.1 Treatments Administered

During the screening period, patients will be allowed to take study allocated Gaviscon® (rescue medication) up to 2 doses per day. During the screening period and the treatment phase, patients are prohibited from taking prescription or OTC medications for the purpose of eradicating *H. pylori* or the treatment of heartburn including all antacid medications. At Visit 2 (Randomisation Visit), patients will be randomised to receive either Zegerid® suspension 20 mg with over-encapsulated placebo capsule or Losec® capsule over-encapsulated with placebo suspension in a 1:1 ratio.

Patients will be provided with Gaviscon® (rescue medication) as separate packs at Visit 1 (Screening Visit) and Visit 2 (Randomisation Visit). Gaviscon® (rescue medication) should only be used, if the patient experiences further episodes of heartburn after an initial episode of heartburn for that particular day (at least 3 hours after the initial intake of randomised study medication), where randomised study medication has been administered and recorded.

9.2 Identity of Investigational Products

9.2.1 Active Study Medication

Drug:	Zegerid®
Ingredients:	Omeprazole, sodium bicarbonate, xylitol, sucrose, sucralose, xanthan gum and flavourings
Formulation:	Powder for oral suspension
Strengths:	20 mg Zegerid®
Posology:	3 x Single doses of 20 mg Zegerid® suspension

9.2.2 Comparator Study Medication

Drug:	Losec®
Ingredients:	Omeprazole, mannitol, hyprolose, cellulose microcrystalline, anhydrous lactose, sodium lauril sulphate, disodium hydrogen phosphate dihydrate, hypromellose, methacrylic acid copolymer, macrogol, colours E171 and E172, gelatine and magnesium stearate

Formulation:	Capsule for oral intake<br>Over-encapsulated capsule (2 piece-hard gelatine, size 00, Swedish orange opaque, microcrystalline cellulose backfill) for oral intake
Strengths:	20 mg Losec®
Posology:	3 x Single doses of 20 mg Losec® capsule.

9.2.3 Placebo Medication

Drug:	Placebo (oral suspension / over-encapsulated)
Ingredients:	Sucralose, xanthan gum, sodium chloride, maltodextrin, xylitol, peppermint (flavour), artificial peach powder (flavour), starch (corn), sugar powder (NF1 and NF2)
Formulation:	Powder for oral suspension<br>Over-encapsulated capsule (2 piece-hard gelatine, size 00, Swedish orange opaque, microcrystalline cellulose backfill) for oral intake
Strengths:	20 mg
Posology:	3 x Single doses of placebo capsule / 3 x single doses of placebo suspension.

9.2.4 Antacid Rescue Medication

Drug:	Gaviscon®
Ingredients:	Sodium alginate, sodium bicarbonate, calcium carbonate, methyl and propyl hydroxybenzoates and sodium saccharin
Formulation:	Chewable tablets for oral intake
Posology:	32 x Single doses of Gaviscon® Tablets during randomised treatment period<br>16 x Single doses of Gaviscon® Tablets during screening period.

9.3 Labelling and Packaging

Study drugs will be packed and labelled according to pertinent regulations by Norgine Ltd. (for full labelling, refer to TMF and IMPD).

9.4 Dispensing and Storage

The study medication supplied by Norgine Ltd. is to be used exclusively in this clinical study according to the instructions of this protocol.

The Investigator or designee must confirm the receipt of the study medication with his/her signature. A copy of this receipt must be kept by the Investigator or designee and another copy will be stored in the TMF. Study medication must be stored in securely locked areas not generally accessible until dispensed to the patients. The study medication will only be accessible to those persons authorised by the Investigator or designee to dispense study medication. The study medication must be stored at room temperature (not to exceed 25°C) in a dry place.

The Investigator or designee is responsible for the dispensing of study medication and Gaviscon® (rescue medication).

9.5 Accountability for the Study Medication

The returned unused study medication will be accounted for by the Investigator and returned to Norgine Ltd. for destruction. Each application of study medication per patient will be fully documented on drug accountability forms. The Investigator is responsible for the accountability of all used and unused study medication.

9.6 Randomisation

Patients are to be randomised to treatment if they satisfy all the inclusion criteria and are not precluded from participation by any of the exclusion criteria. Patients must also comply with screening period requirements.

Each patient will receive a unique patient number at the Visit 1 (Screening Visit). If the patient is found to be eligible for study participation at the Visit 2 (Randomisation Visit) he/she will be assigned a random number and will receive the study medication with the corresponding number. In case of a withdrawal, the patient's random number will not be reassigned to another patient.

The randomisation list will be produced by a statistician of Premier Research Group Ltd. who is not involved in the conduct or analysis of the study using the SAS system for Windows (SAS Institute Inc, Cary, North Carolina, United States of America [USA]). The randomisation schedule will then be made available to Premier Research IWRS (Interactive Web Response System) and sent to Norgine Ltd. drug packaging group.

9.7 Blinding

This is a double-blind, double-dummy randomised study with both Zegerid® and Losec® being provided in the same packaging and labelling in order to maintain blinding.

No person involved in conducting the study will have access to the randomisation code before the blind is officially broken. However, in the case of an emergency that requires the investigator to be unblinded, the investigator can obtain the randomisation code via Premier Research IWRS. Only users with specified access via a user identification and password assigned by Premier Research will be able to unblind the patient. The Norgine Drug Safety Group representative will also have specified access and can determine which treatment for a particular patient was given by accessing Premier Research IWRS. The Investigator or Norgine Ltd. will state the reason why the code was broken as part of the IWRS process.

9.8 Treatment Compliance

Patients will be provided with an Electronic Diary (e-diary), which will be used to record details of when each dose of randomised study medication has been taken. The data captured on the e-diary will be reviewed using a validated database, which will allow patients to upload their data and for Investigators to download the recorded data.

10 PRIOR AND CONCOMITANT MEDICATION AND OTHER RESTRICTIONS

10.1 Prior Medication

All prior medications taken within 28 days of Visit 1 (Screening Visit) are to be recorded.

10.2 Concomitant Medication

All concomitant medications taken during the study will be recorded. Excluded medications during study participation include:

1. Theophylline, bismuth salts, warfarin, phenytoin, barbiturates, antineoplastic agents, erythromycin, clarithromycin, or sucralfate (also within last 2 weeks before first dose of study medication)
2. Any medication for the purpose of the eradication of *H. pylori* (also during the last 28 days before the start of the study)
3. Systemic glucocorticoids or non-steroidal anti-inflammatory drugs (NSAIDs) including COX-2-inhibitors (≥3 consecutive days per week, during the last 28 days before the start of the study and as treatment requirement for study phase); except regular intake of enteric coated aspirin dosages up to 150 mg/d
4. Concomitant medications that rely on the presence of gastric acid for optimal absorption (e.g. ketoconazole).

10.3 Other Restrictions

The Investigator or designee will request that patients abstain from:

- Alcohol consumption throughout screening period and randomised treatment period.

11 CONDUCT OF THE STUDY AND METHODS OF ASSESSMENT

Patients will be sequentially assigned a screening number starting 0001 and prefixed with the study site number at Visit 1 (Screening Visit). Each patient will be screened for suitability during Visit 1 from the proposed first administration date. Patients will initially be instructed to record daily episodes of heartburn using an e-diary (and the Likert severity scale) as provided, and to only use Gaviscon® (rescue medication) provided by the Investigator. All patients will undergo the following assessments to check suitability against the inclusion/exclusion criteria:

Visit 1 – Screening Visit

- Informed Consent/Patient Information
- Review of Inclusion/Exclusion Criteria
- Demographics/Medical History
- Vital Signs
- Clinical Laboratory Tests (including clinical chemistry, haematology and urinalysis)
- Physical Examination
- Concomitant Medication
- Concomitant Antacids
- Pregnancy Test
- Instructions and Training on competent use of e-diaries.

After satisfactory completion of each patient's screening examination, dates for the return to the site will be proposed assuming that all outstanding tests do not preclude the patient receiving randomised study medication. Each patient will be reminded of the inclusion and exclusion criterion prior to attending the clinic (Sections 8.1 and 8.2).

Visit 2 – Randomisation Visit (Day 1)

After confirming all inclusion/exclusion criteria requirements have been met, the treatment number assignment will occur on Day 1. Each patient will be randomised to one of the following treatment arms; Zegerid® 20 mg suspension plus over-encapsulated placebo capsule or Losec® 20 mg capsule over-encapsulated with placebo suspension. Each patient will receive 3 days use of randomised study medication and 14 days use of Gaviscon® (rescue medication). Study medication will be self-administered only when the patient experiences their first episode of heartburn

for that particular day. Further episodes of heartburn on the same day should be treated using study allocated Gaviscon® (rescue medication) for a maximum of 2 doses per day. Any physical changes from the physical assessment at Visit 1 will be noted in the e-CRF.

The following assessments will be performed:

- Physical Examination
- Review of Inclusion/Exclusion Criteria
- Review of Concomitant Medication(s)
- Review of Concomitant Antacids
- Review of AE(s)
- Review of e-diary use and compliance.

Visit 3 – End of Study Visit (Day 15)

The patient will be required to attend the site at Day 15 for an End of Study Visit. This visit will entail the following assessments:

- Physical Examination
- Concomitant Medications
- Concomitant Antacids
- AE(s)
- Study Medication Accountability
- Review e-diary compliance.

Any physical changes from the physical assessment at Visit 1 will be noted in the e-CRF.

11.1 Demographic Data and Medical History

Demographic information will be collected for all patients at Visit 1 (Screening Visit) and will include ethnic group, age, date of birth (month/year), gender, weight and height.

Medical history will be collected for all patients at Visit 1 (Screening Visit) and will include details regarding all illnesses and surgery, any kind of allergies, dates of onset, and whether the condition currently persists. Childhood illnesses are not to be documented unless they are considered to be relevant for the study indication or could potentially give rise to an AE.

All records of patient medical history will be coded using MedDRA, Version 13.0 or higher.

11.2 Electronic Diary

Patients will record data using an e-diary device. Patient-reported data will be received electronically from CRF Health and loaded into the study database via a validated path. The data will be presented back to the Investigational sites via the CRH health portal.

An e-diary service provider (CRF Health) will produce the software accordingly to the requirements of the study and will provide instruction manuals for users (patients) and trainers (site staff).

All patients will be provided with instruction cards to assist in the use of the e-diaries after they have been trained by the Investigator at Visit 1 (Screening Visit). All patients will be assessed on competent e-diary use at Visit 1.

11.3 Electronic Data Capture

Syne qua non Ltd. will supply the Investigator sites with access to the EDC system that has been fully validated and conforms to 21 CFR Part 11 requirements. Syne qua non Ltd. personnel will train designated investigational staff on the use of the EDC system including any study-specific details, and a user manual will be provided. Investigational staff will not be given access to the EDC system until they have been trained and assessed as competent to use the system.

Designated investigational staff will enter the data required by the protocol into the e-CRFs. Automatic data validation will check for data discrepancies in the e-CRFs and, by generating appropriate error messages, allow modification or verification of the entered data by the investigational staff. The Principal investigator or Sub-investigator must certify that the data are complete and accurate within the system, following Source Data Verification (SDV) of selected data.

11.4 Safety Assessments

11.4.1 Adverse Events

The documentation of AEs is described in Sections 14.5 and 14.6.

11.4.2 Clinical Laboratory Tests

Blood samples or urine samples will be taken at Visit 1 (Screening Visit) to confirm patient eligibility for the study.

All blood samples for the safety laboratory tests will be evaluated at the local study site laboratory. Sample collection will be carried out according to each local laboratory practice.

Each blood sample will be 20 mL or less in volume. Any blood results falling outside of the laboratory normal range will be flagged and reviewed by the Investigator. If there are any abnormalities that the Investigator considers to be clinically significant, the patient should not be included in the study. If additional laboratory tests are carried out during the study that, in the Investigator's opinion, show clinically significant or pathological changes during or after termination of the treatment, these should be reported as AEs and have to be followed with appropriate medical care, even after termination of the study, until normal or baseline values are reached and the condition has stabilised or a non-study medication cause has been identified. The following parameters will be determined as a minimum[4]:

Biochemistry:	Sodium, Potassium, Urea, Creatinine, Bicarbonate, Chloride, Alanine Transaminase (ALT), Asparate Aminotransferase (AST), Alkaline Phosphate (ALP), Albumin, Total Protein, Bilirubin
Haematology:	Haemoglobin, Haematocrit, Erythrocytes (RBC), Mean Cell Haemoglobin (MCH), Leukocytes (WBC), Differential Count, Platelet

Urine samples will be collected from women of child-bearing potential and a urine pregnancy test will be carried out at Visit 1 (Screening Visit). Any woman with a positive pregnancy test result must be withdrawn from the study. The following parameters will be determined as a minimum:

Urinalysis:	Glucose, Protein, Creatinine, Sodium, Potassium, Bilirubin, Urine Specific Gravity, Erythrocytes, Leukocytes

11.4.3 Physical Examination

A full physical examination will be performed at Visit 1 (Screening Visit), with any changes from this assessment then being noted in the e-CRF at the subsequent study visits (Visits 2 and 3).

The following organ systems will be examined: cardiovascular, respiratory, abdominal, genitourinary, gastrointestinal, musculoskeletal, neurological and dermatological.

[4] Absolute values will be calculated

11.4.4 Vital Signs

Blood pressure (BP: Systolic [SBP]/Diastolic [DBP]) and pulse rate (PR) will be measured at the following visits:

- Screening Visit –Visit 1
- Randomisation Visit – Visit 2.

Body temperature will be documented at Visit 1 (Screening Visit) and Visit 2 (Randomisation Visit): temperature will be recorded in degree Celsius (°C). Time and date of vital signs assessment will be fully documented.

12 DATA COLLECTION AND ENTRY

The Sponsor of this study is Norgine Ltd. and the Contract Research Organisation (CRO) conducting this study for Norgine Ltd. is Premier Research Group Ltd.

To ensure consistency between study sites in this study, an Investigators meeting will be held to ensure all Investigators are familiar with the protocol, the AE reporting process, unblinding procedures and their responsibilities under GCP requirements. In addition, all study sites will be regularly monitored to ensure competent use of the e-CRFs and e-diaries, and to ensure the study is being conducted according to ICH-GCP (see Section 12.1).

12.1 Monitoring

The study will be monitored at regular intervals during the enrolment period. The frequency of monitoring visits will be determined by the rate of patient recruitment. The following will be reviewed at these visits:

- Responsibilities of the Investigator and the study site under GCP requirements
- Compliance with the protocol
- Consent procedure
- SDV
- Procedures for AEs
- Storage and accountability of study medication.

The purpose of SDV is to verify, so far as is possible, that the information in the e-CRF reflects the data recorded in the patient's medical records. SDV will be performed with due regard for patient confidentiality. SDV will be undertaken on an ongoing basis as part of the monitoring visits. Direct access to the source documents will be required. The monitor will make a direct comparison with data entered in the patient e-CRFs, patient e-diaries and the patient's medical records.

The Investigator must permit the monitor, Norgine Ltd's internal auditors and representatives from the Regulatory Authorities to inspect all study-related documents and pertinent hospital or medical records for confirmation of data contained within the e-CRFs.

12.2 Data Management

Syne qua non Ltd will be responsible for activities associated with the data management of this study, setting up a relevant validated database and data transfer mechanisms, along with appropriate validation of data and resolution of data queries.

12.2.1 Data Entry

Study-specific data will be collected directly from sites using an EDC system provided by Syne qua non Ltd. For each patient enrolled, an e-CRF will be completed at the study sites and signed by the Investigator within a reasonable time period after data collection.

Patient e-diary data will be received electronically from CRF Health and loaded into the study database via a validated path.

Syne qua non Ltd. will supply the study sites with access to the Electronic Data Capture (EDC) system that has been fully validated and conforms to 21 CFR Part 11 requirements. Investigational staff will not be given access to the EDC system until they have been trained and assessed as competent to use the system.

Designated investigational staff will enter the data required by the protocol into the e-CRFs. Automatic data validation will check for data discrepancies in the e-CRFs and, by generating appropriate error messages, allow modification or verification of the entered data by the investigational staff.

Monitoring staff will review the e-CRFs entered by investigational staff for completeness and accuracy and instruct the investigational staff to make any required corrections or additions. Queries will be raised to the investigational staff within the EDC system. Designated investigational staff will be required to respond to the query and make any necessary changes to the data, and close queries after appropriate action has been taken.

The Investigator must review the e-CRFs for completeness and accuracy and must electronically sign and date the appropriate e-CRFs. The investigator must retain full responsibility for the accuracy and authenticity of all data entered on the e-CRFs.

After completion of monitoring activities and Investigator sign off, data management activities will be undertaken by Syne qua non Ltd., who will access the data in the system, and run appropriate validation checks. Data queries raised during the data management process will be issued to the investigational staff for resolution within the EDC system. Those data, where appropriate, will go through the monitoring and investigator sign-off process.

All actions within the system are captured within the audit trail.

After all data have been entered, validated and signed off as complete the database will be locked.

12.2.2 Data Coding

For data coding, the following thesauri will be used:

- AEs, medical history and concurrent illnesses: MedDRA, Version 13.1 or higher. Coding will take place using current Lowest Level Terms in accordance with pre-determined coding conventions
- Prior and concomitant medication: World Health Organisation (WHO)-Drug Dictionary, Standard Version September 2010.

12.2.3 Data Validation

Visual and computerised methods of data validation will be applied in order to ensure accurate, consistent and reliable data for the subsequent statistical analysis. These procedures aim to detect out-of-range values, contradictory data, abnormal evolutions over time, and possible undetected protocol violations (eligibility criteria, time and medication compliance, etc.).

12.2.4 Disclosure of Randomisation Code/Database Lock

The database will be locked in order to protect write access after the following pre-conditions are fulfilled:

- All data are entered in the database
- Decisions have been made and agreed to as to the identities of all protocol violators
- Decisions have been made and agreed as to inclusion in the analysis populations
- Written authorisation from Norgine Ltd.

The randomisation code for this study will not be revealed until the following pre-conditions are fulfilled:

- The database is officially locked
- Written authorisation from the Norgine Ltd. is obtained.

13 STATISTICAL ANALYSIS

13.1 Primary Endpoint

The primary efficacy parameter is the time to sustained response, which is defined as a reduction of severity of heartburn associated with gastroesophageal reflux disease (GERD) by 3 points or more on a 9-point Likert severity scale, which is sustained for 45 minutes or more.

13.2 Secondary Endpoints

Key secondary:

1. The time to sustained partial response, defined as a reduction of 2 or more points on the 9-point Likert severity scale, which is sustained for 45 minutes or more.
2. The time to sustained total response, defined as zero severity (no heartburn) on the 9-point Likert severity scale, which is sustained for 45 minutes or more.
3. The proportion of patients who have achieved sustained response, sustained partial relief or sustained total relief by 45, 60 and 90 minutes.

Secondary:

1. The severity of heartburn associated with GERD and change in severity from pre-dose at all time points.
2. The proportion of patients who have achieved sustained response, sustained partial response or sustained total relief at all other time points.
3. The proportion of patients with total relief (defined as zero severity on the 9-point Likert severity scale) of heartburn associated with GERD at all time points.
4. The proportion of patients with response (defined as a reduction in the severity of heartburn from pre-dose of 3 or more points on the 9-point Likert severity scale) at all time points.
5. The proportion of patients with at least partial response (defined as a reduction in the severity of heartburn from pre-dose of 2 or more points on the 9-point Likert severity scale) at all time points.
6. The area under the severity-time curve (AUC) will be calculated for the for the following time periods; 0-60, 0-120 and 0-180 minutes.
7. The usage of Gaviscon® (rescue medication) over the 14 day randomised treatment period.
8. Safety and tolerability.

13.3 Definition of Study Populations for Analysis

13.3.1 Safety Population

The Safety population will consist of all randomised patients who received at least one dose of randomised study medication during the course of the study.

13.3.2 Modified Intention-to-Treat Population

The Modified Intention-To-Treat (mITT) population will consist of all patients in the Safety population who are evaluable (recorded data for at least one evaluable episode of heartburn).

13.3.3 Per Protocol Population

The Per Protocol (PP) population will consist of all patients included in the mITT population but will exclude the following:

- Patients violating any inclusion/exclusion criterion
- Patients with major protocol violations (e.g., poor compliance, incorrect completion of e-diaries) in a sufficiently serious manner to warrant exclusion.

13.4 Determination of Sample Size

Zegerid® has an expected median time to response of 45 minutes compared to a median time to response for Losec® of 75 minutes. With 90 % power and a two-sided level of significance of 0.05 (5%), 97 patients are required per treatment group.

The software nQuery Advisor v6.1 was used for the calculation and a log-rank test assumed.

13.5 Statistical Methods

13.5.1 Descriptive Statistics

For dichotomous and categorical variables, absolute and relative frequencies (counts and percents) will be calculated. For continuous variables, comprehensive data summaries will be presented with sample characteristics (n, arithmetic mean, standard deviation [SD], minimum, lower quartile, median, upper quartile and maximum) for all continuous variables by treatment. Where data are collected over time, both the observed data and the change from the run-in period will be summarised at each time point.

13.5.2 Analysis of Baseline Data

Baseline characteristics will be summarised by treatment for each population. There will be no statistical testing of the comparability of the populations.

13.5.3 Analysis of Efficacy

The primary efficacy hypothesis will be evaluated in a confirmatory test.

Each patient will have data from between 1 and 3 episodes of heartburn that have been treated with the study medication. The median will be used to give an average severity of heartburn for each patient at each time point.

An evaluable episode will be one with a baseline severity of level 4 or more and no more than 1 missing time point in the 3 hour period. If rescue medication is taken in the 3 hour period, the last observation carried forward method will be used using the severity recorded when the rescue medication was taken and the episode will be considered evaluable. The patient will be considered a non-responder at all later time points.

A patient will be evaluable if they have at least one evaluable episode.

All efficacy analyses will use the mITT population and the PP population.

Primary Efficacy Analysis

The primary efficacy parameter is the time to sustained response, which is defined a reduction of 3 points or more on the 9-point Likert severity scale which is sustained for 45 minutes or more. This will be defined using the average heartburn severity at each time point. Patients who do not achieve sustained response will be censored at the last evaluable time point. The time to the start of this response will be shown in each treatment group using a Kaplan-Meier curve. The median time and the 95% confidence interval will be presented for each treatment. Differences between the two treatments will be tested at a two-sided significance level of 0.05 (5%) using a Cox regression model, including a centre effect. A 95% confidence interval will be presented for the treatment hazards ratio.

Secondary Efficacy Analysis

Key Secondary efficacy parameters:
The time to sustained partial response and the time to sustained total relief will be analysed in the same way as the primary efficacy parameter.

The proportion of patients who have achieved sustained response, sustained partial response or sustained total relief in each treatment group by 45, 60 and 90 minutes will be presented from the Kaplan-Meier analysis. A 95% confidence interval will be represented for the treatment difference at each time point.

Secondary efficacy parameters:
The severity of heartburn and change from baseline will be summarised with descriptive statistics for each treatment at each time point.

The AUCs will be summarised with descriptive statistics for each treatment at each time point.

The proportion of patients who have achieved sustained response, sustained partial response or sustained total relief in each treatment group at all other time points will be presented from the Kaplan-Meier analysis.

The proportion of patients who have achieved response, partial response or total relief in each treatment group at all time points will be summarised by treatment in frequency tables.

The total use of Gaviscon® (rescue medication) over the 14 day randomised treatment period will be summarised as the number of doses taken. The proportion of days where Gaviscon® (rescue medication) was taken and the average number of doses taken per day will be presented.

No testing is planned for any of these secondary parameters, although further exploratory analyses may be added. These will be described in the Statistical Analysis Plan.

13.5.4 Planned Safety Analyses

All safety analyses will use the Safety population.

The primary safety parameter is the occurrence of SAEs and AEs. All data will be summarised within each treatment group. All SAEs and AEs will be listed using coding for System Organ Class and Preferred Term (using the MedDRA version 13.0 or higher). Only treatment-emergent AEs will be included in the analysis.

SAEs and AEs will be summarised as follows:

- Number and percentage of patients with AEs classified by System Organ Class and Preferred Term
- Number and percentage of patients with AEs by strongest relationship to randomised study medication, System Organ Class and Preferred Term
- Number and percentage of patients with AEs by maximum severity, System Organ Class and Preferred Term
- Number and percentage of patients with serious adverse events (SAEs) classified by System Organ Class and Preferred Term

- Number and percentage of patients with drug-related SAEs classified by System Organ Class and Preferred Term.

13.5.5 Interim Analyses

No interim analysis is planned for this study.

14 ADVERSE EVENTS, SERIOUS ADVERSE EVENTS AND REPORTING

An AE is the development of an undesirable medical condition or the deterioration of a pre-existing medical condition following or during exposure to a pharmaceutical product, whether or not considered causally related to the treatment. An undesirable medical condition can be symptoms (e.g. nausea, chest pain), signs (e.g. tachycardia, enlarged liver) or the abnormal results of an investigation (e.g. laboratory findings, electrocardiogram [ECG]). In clinical studies, an AE can include an undesirable medical condition occurring at any time, including run-in or washout periods, even if no study treatment has been administered.

This definition includes events occurring from the time of the patient giving informed consent until the end of the study.

14.1 Disease Progression

This study plans to recruit patients with a history of heartburn associated with GERD. Sequelae, progression or exacerbation of any underlying condition either related or unrelated to the patient's episodes of heartburn identified during the medical history, physical examination or during the study will be classified as an AE.

14.2 Categorisation of Adverse Events

14.2.1 Intensity Classification

AEs will be classified as mild, moderate or severe according to the following criteria:

Mild: Symptoms do not alter the patient's normal functioning.

Moderate: Symptoms produce some degree of impairment to function, but are not hazardous, uncomfortable or embarrassing to the patient.

Severe: Symptoms definitely hazardous to well-being, significant impairment of function or incapacitation.

14.2.2 Causality Classification

The relationship of an AE to study medication will be classified according to the following:

Probable: A reaction that: follows a reasonable temporal sequence from administration of the drug, follows a known or expected response pattern to the suspected drug; is confirmed by improvement on stopping or reducing the dosage of the drug or could not be reasonably explained by the known characteristics of the patient's clinical state.

Possible: A reaction that follows a reasonable temporal sequence from administration of the drug and that follows a known or expected response pattern to the suspected drug but that could readily have been produced by a number of other factors.

Not related: Any event for which there is sufficient and conclusive information that the event is not related to the study drug.

14.2.3 Treatment-Emergent Adverse Events

An AE is defined as treatment-emergent, if, and only if, first onset or worsening is after first intake of study medication. Additionally, any AE thought to be related to study treatment is defined as treatment emergent, whenever the first onset of the event.

14.2.4 Assessment of Expectedness

The expectedness of an AE or reaction shall be determined by Norgine Ltd according to the most recent Investigator Brochure for Zegerid® and Summary of Product Characteristics (SmPC) for omeprazole.

14.2.5 Laboratory Test Abnormalities

Any abnormalities that the Investigator considers to be clinically significant, the patient should not be included in the study. If additional laboratory tests are carried out during the study that, in the Investigator's opinion, shows the following:

- Clinically significant or pathological changes during or after termination of the treatment,
- Results in a change in study medication schedule of administration (change in dosage, delay in administration, study medication discontinuation),
- Requires intervention or a diagnosis evaluation to assess the risk to the patient.

14.2.6 Abnormal Physical Examination Findings

Abnormal physical examination findings post dosing will be recorded as AEs if the Investigator considers they are clinically significant.

14.2.7 Other Investigation Abnormal Findings

Abnormal test findings will be recorded as AEs if the Investigator considers they are clinically significant. They may include but are not limited to ECG changes, factors that require changes in study medication dosage or administration schedule, discontinuation of study medication or factors that require intervention or diagnostic evaluation to assess the risk to the patient.

14.3 Recording and Follow-up of Adverse Events

The patients will be instructed to report AEs to the Investigators. Standardised AE questioning will be conducted at screening and throughout the study during visits. Patients will be requested to report any severe or serious adverse events to the Investigator as soon as possible.

Any AEs observed or reported by a patient and/or staff will also be recorded in the e-CRF. The Investigator will review results from the physical examinations. All new and aggravated findings, as compared with baseline, must be identified and recorded in the AE section of the e-CRF. These will be considered AEs.

AEs already recorded and designated as 'continuing' should be reviewed at each subsequent assessment.

For all AEs, the Investigator must obtain adequate information to determine the outcome of the AE and to assess whether it meets the criteria for classification as an SAE. For all AEs, sufficient information should be obtained by the Investigator to determine the causality of the AE (i.e. study medication or other illness). The Investigator is required to assess causality and record that assessment on the e-CRF. Follow-up of the AE, after the date of therapy discontinuation, is required if the AE or its sequelae persist. All AEs will be followed up until the outcome is determined or stabilised at a level acceptable to the Investigator or for at least for 4 weeks after conclusion of the clinical conduct of the study. All serious AEs will be followed up until the outcome is determined or up to 3 months after conclusion of the clinical conduct of the study. The findings of these follow-up investigations will be communicated to Norgine Ltd.

14.4 Serious Adverse Events

14.4.1 Definition

All SAEs (as defined below) regardless of treatment group or suspected relationship to study medication must be reported immediately (within 24 hours of the Investigator's knowledge of the event) to Norgine's Drug Safety Group, Fax: +44 (0) 1895 453 732. If the immediate report is submitted by telephone, this must be followed by detailed written reports using the SAE report form within 24 hours of the Investigator's

knowledge of the event.

An SAE is any AE that:

1. Results in death
2. Is considered life threatening, which is any event that places the patient at immediate risk of death from the reaction as it occurred. It does not include a reaction that, had it occurred in a more severe form, might have caused death
3. Results in in-patient hospitalisation or prolongation of existing hospitalisation, excluding admission for social or administrative reasons
4. Results in a persistent or significant disability/incapacity, where disability is a substantial disruption of a person's ability to conduct normal life functions
5. Results in congenital anomaly/birth defect in the offspring of a patient who received study medication
6. Is an important medical event that may not result in death, be life-threatening, or require hospitalisation when, based upon appropriate medical judgement, may jeopardise the patient and may require medical or surgical intervention to prevent one of the outcomes listed in this definition. Examples of such medical events include allergic bronchospasm requiring intensive treatment in an emergency room or at home, blood dyscrasias or convulsions that do not result in in-patient hospitalisation, or the development of drug dependency or drug abuse.

Regardless of the above criteria, any additional AE that Norgine Ltd. or an Investigator considers serious should be immediately reported to Norgine Ltd. and will be entered into Norgine's SAEs database system.

- Hospitalisation is defined as any in-patient admission (even if less than 24 hours). For in-patients, hospitalisation also includes transfer within the hospital to an acute/intensive care in-patient unit.
- Prolongation of hospitalisation is defined as any extension of an in-patient hospitalisation beyond the stay anticipated/required in relation to the original reason for the initial admission, as determined by the Investigator or treating physician. For protocol-specified hospitalisation in clinical studies, prolongation is defined as any extension beyond the length of stay described in the protocol. Prolongation in the absence of a precipitating, treatment-emergent, clinical AE (i.e. not associated with the development of a new AE or worsening of a pre-existing condition) may meet criteria for "seriousness" but is not an adverse experience and thus is not patient to immediate reporting to Norgine Ltd.

Pre-planned or elective treatments/surgical procedures should be noted in the patient's screening documentation. Hospitalisation for a pre-planned or elective treatment/surgical procedure should not be reported as an SAE unless there are

complications or sequelae which meet the criteria for seriousness described above.

14.4.2 Reporting Requirements

Any SAE must be reported immediately (within 24 hours), independent of the circumstances or suspected cause, if it occurs or comes to the attention of the Investigator at any time during the study period.

SAEs must be reported to:

Norgine Ltd.
Norgine House, Widewater Place, Moorhall Road,
Harefield, Uxbridge, UB9 6NS, United Kingdom

Tel: +44 (0) 1895 826 776 ; Fax: +44 (0) 1895 453 732

Norgine Ltd's Medical Officer can be contacted on Tel: +44 (0) 1895 453 725; Fax: +44 (0) 1895 453 729; E-mail: hgruss@norgine.com.

If the Norgine Ltd. Representative cannot be reached, the following dedicated 24-hour emergency number can be used to contact a Norgine Trial Physician (NTP) for resolution of urgent safety questions: +44 (0) 1748 828 787.

Any SAE with a suspected causal relationship to study medication occurring at any other time after completion of the study must be promptly reported.

14.4.3 Mandatory Information for Reporting an SAE

The following information is the minimum that must be provided to Norgine Ltd. Pharmacovigilance contact within 24 hours for each SAE:

- Study number
- Site number
- Patient number
- AE
- Investigator's name and contact details

The additional information included in the SAE form must be provided to Norgine Ltd. or representative as soon as it is available. Upon receipt of the initial report, Norgine Ltd. will ask for the Investigator's causality assessment if it was not provided with the initial report.

The Investigator should report a diagnosis or a syndrome rather than individual signs or

symptoms. The Investigator should also try to separate a primary AE considered as the foremost untoward medical occurrence from secondary AEs which occurred as complications.

14.4.4 Reporting Exemptions

There are no reporting exemptions.

14.5 Pregnancy

Pregnancy itself is not regarded as an AE unless there is a suspicion that study medication has interfered with a contraceptive method. If pregnancy occurs during the study, the outcome of the pregnancy will then need to be collected post-study and it will be necessary to discontinue treatment with study medication and withdraw the patient.

Information regarding pregnancies must be collected on the AE page of the e-CRF and the Standard Pregnancy Outcome Report Form (available upon request), including pregnancies with normal progress and outcome. A Standard Pregnancy Outcome Report Form must be completed by the Investigator and provided to Global Pharmacovigilance within 24 hours of the knowledge of the pregnancy in any study patient.

Investigators must instruct all female patients to inform them immediately should they become pregnant during, or within 28 days of the study. The Investigator should counsel the patient; discuss the risks of continuing with the pregnancy and the possible effects on the foetus. Monitoring of the patient should continue until conclusion of the pregnancy, which may involve follow-up after the patient's involvement in the study, has ended. In cases of pregnancy in the partners of male patients, details will be collected using Norgine's standard process.

14.6 Deaths

All AEs resulting in death either during the study period or within 28 Days after the last dose of study medication must be reported as an SAE within 24 hours of the Investigator's knowledge of the event.

The convention for recording death is as follows:

- AE term: lead cause of death (e.g. multiple organ failure, pneumonia, myocardial infarction)
- Outcome: fatal.

The only exception is if the cause of death is unknown (i.e. sudden or unexplained

death), in which case the AE term may be 'Death' or 'Sudden death'.

14.7 Discontinuation/Withdrawal due to Adverse Events/Serious Adverse Events

Discontinuation/withdrawal due to AEs should be distinguished from discontinuation/ withdrawal for other reasons.

If study medication is discontinued due to a SAE it must be reported immediately to Norgine Ltd's Representative. In all cases the Investigator must ensure the patient receives appropriate medical follow-up.

14.8 Reporting to Competent Authorities/IECs/IRBs/Other Investigators

Norgine Ltd. will submit reports of Suspected Unexpected Serious Adverse Reactions (SUSARs) occurring during the study to the Competent Authorities (CA), IECs, IRBs and other Investigators concerned by the study medication. Reporting will be done in accordance with the applicable regulatory requirements and Norgine Ltd. procedures.

14.9 Safety Laboratory

The following safety parameters will be assessed: Type and frequency of AEs, laboratory parameters (haematology, biochemistry and urinalysis), vital signs and physical examination. See Section 11.3.

15 ETHICAL CONSIDERATIONS AND INSURANCE

15.1 Patient Information and Informed Consent

Prior to study start, the patient will receive a full explanation of the study, study medication, and all possible side effects in writing and additional verbal explanations. All patients must fully understand the explanations before signing the informed written consent. The consent form has to be signed and dated personally by the patient and the Investigator, which will be stored with the Investigator for a period of 15 years. The patient will receive a copy of the signed consent form. The monitor will check the consent form for all screened patients to ensure that consent has been granted prior to the conduct of any study specific procedures.

15.2 Ethics Committee(s)

Prior to study start, the protocol and the informed consent form (as a minimum) will be submitted to the relevant IEC(s). The study will be registered with www.clinicaltrials.gov.

15.3 Amendments to the Protocol

Changes to the protocol which may have an influence on the scientific rationale, the risk/benefit ratio, health aspects of the healthy patients or constitute a substantial change to the original application will require re-submission to the Ethics Committee. Patients who are actively participating in the study will be re-consented if required. The signed amendments will be attached to the protocols in use. The amendments will replace the relevant sections in the original protocol.

Administrative or technical changes of the protocol which have no influence on the health aspects of the patients will also require a change to the protocol. This change of the protocol will be submitted to the IEC(s) and CA(s) for notification only and will be attached to the protocol in use.

15.4 Finance and Insurance

Details on finance will be outlined in a separate agreement between the Investigator and Premier Research Group Ltd. as agreed and approved by Norgine Ltd.

The patient will be informed of the insurance coverage and the address of the insurance company. According to the insurance conditions the policy holder or authorised third party have to inform the patient with regard to the patient's insurance and the relevant obligations, especially concerning the obligations of the patients as:

- The patients must not have alternative medical treatment (except in cases of emergency or approved by the Investigator)
- An impairment of health, which might occur due to the clinical trial, must be forwarded to the insurance company immediately, via Norgine Ltd.
- The notification of Norgine Ltd. should be handled by the Investigator.

15.5 Regulatory Requirements

This study will be performed according to the principles of the Declaration of Helsinki (Seoul, October 2008).

All physicians involved in the clinical study are obliged to conduct and carry out the study according to the Guidelines of ICH-GCP, EU Clinical Trials Directive and the Declaration of Helsinki. Similarly, all employees from Norgine Ltd., involved in the clinical study, will strictly adhere to ICH-GCP and all requirements stated in the EU Clinical Trials Directive.

16 GENERAL OBLIGATIONS, AGREEMENTS AND ORGANISATION

16.1 Investigators Brochure

The study site will be informed of the non-clinical and clinical medical knowledge by Norgine Ltd. As soon as new results are available the Investigators will be updated with respect to new safety information.

16.2 Data Protection and Confidentiality of the Investigator

The name of the patient and any other confidential data will be protected by the Investigator. Should it be required during the conduct of the study to identify the name, this will be done only following the legal obligation of the physician. The Investigator will particularly pay attention that e-CRFs or additional documents handed over to Norgine Ltd. obscure patient names.

16.3 Electronic CRFs and Handling

Premier Research Group Ltd. will supply the study sites with e-CRFs, and an Investigator Site file.

Data will be collected directly from sites using an EDC system provided by Syne qua non Ltd. For each patient enrolled, an e-CRF will be completed at the investigational sites and signed by the Principal Investigator within a reasonable time period after data collection.

The Investigator must review the e-CRFs for completeness and accuracy and must electronically sign and date the appropriate e-CRFs. The Investigator must retain full responsibility for the accuracy and authenticity of all data entered on the e-CRFs.

After Investigator sign-off, data management activities will be undertaken by Syne qua non Ltd., who will access the data in the system, and run appropriate validation checks. Data queries raised during the data management process will be issued to the investigational staff for resolution within the EDC system. Those data, where appropriate, will go through the monitoring and Investigator sign-off process.

All actions within the system are captured within the audit trail.

After all data have been entered, validated and signed off as complete the database will be locked. After database lock, the Investigator will receive a CD-ROM or paper copies of the patient data for archiving at the investigational site.

All e-CRFs and clinical study data will be stored by Norgine Ltd. for a period of at least 15 years after termination of the study. The patient consent forms and re-identification lists will be archived with the Investigator for at least 15 years after termination of the study.

16.4 Storage of Study Documents and Investigator Site File

The Investigator will be supplied with an Investigator Site File for permanent storage of all documents applicable to this clinical study.

16.5 Confidentiality

All study documentation and e-CRF data must be kept strictly confidential and may not be disclosed to third parties. All members of staff at the study site and within Norgine Ltd. are obliged to comply with this requirement.

16.6 Publication

After completion of the study a report will be written based on the statistical analysis and the final integrated report will be signed by the Principal Investigator and the Vice President of Clinical Development, Norgine Ltd. (as a minimum).

Publication of the results is encouraged after appropriate time for review and written agreement by Norgine Ltd. Norgine Ltd. will check and authorise any manuscripts to be published at least 4 weeks before submission to a journal.

17 QUALITY ASSURANCE/AUDIT

This study may be subject to audit by Norgine Ltd. or their representatives and Regulatory Authorities. These audits may be undertaken to check compliance with the requirements of GCP and can include:

- In-house study file audit
- Audit of computer database
- Audit of Clinical Study Report
- Audit of selected study sites, requiring access to patient medical records, study documentation and facilities, laboratories or pharmacies used for the study.

The study site, facilities and all data (including source data) and documentation will be made available for audit by the Investigator according to the ICH-GCP guidelines. The Investigator agrees to co-operate with the auditor during his/her visit and will be available to supply the auditor with e-CRFs or other files necessary to conduct that audit. Any findings will be strictly confidential.

In the event that a Regulatory Authority informs the Investigator that it intends to conduct an inspection, Norgine Ltd. must be notified immediately.

18 REFERENCES

1. Colin W. Howden, E. David Ballard, K. Koch, Yun Hardiman, A David Mundt, Theresa C. Gautille, Robert G. Bagin. Control of 24-Hour Gastric Acidity After Morning Dosing with Immediate-Release and Delayed –Release Proton Pump Inhibitors in Patients with GERD Symptoms. Gastroenterology, Volume 134, Issue 4, Supplement 1, April 2008, Pages A-174.

2. Jones, T. Patrikios. The Effectiveness of Esomeprazole 40 mg in Patients with Persistent Symptoms of Gastro-oesophageal Reflux Disease following Treatment with a full dose Proton Pump Inhibitor. Int J Clin Pract. December 2008, 62, 12, 1844–1850.

3. R. Jones, H. R. Liker, P. Ducrotté. Relationship between Symptoms, Subjective Well-being and Medication use in Gastro-oesophageal Reflux Disease. Int J Clin Pract. 2007 August; 61(8): 1301–1307.

4. Joseph A. Crawley, Bernard Hamelin and Eileen Gallagher. How satisfied are Chronic Heartburn Sufferers with the Results they get from Prescription Strength Heartburn Medication? Gastroenterology Volume 118, Issue 4, Part 1, April 2000, Page A210.

5. Ronnie Fass, Stephen J. Sontag, Barry Traxler, and Mark Sostek. Treatment of Patients With Persistent Heartburn Symptoms: A Double-Blind, Randomized Trial. Clinical Gastroenterology and Hepatology 2006; 4: 50–56.

6. Heartburn – Topic Overview. WebMD.com, Medical reference from Healthwise, Inc. 01 September 2009

7. Monika Sarker, MD; Sean Hennessey, PharmD, PhD; and Yu-Xiao, MD, MSCE. Proton-Pump Inhibitor Use and the Risk for Community-Acquired Pneumonia. Ann Intern Med. 2008; 149(6): 391-398.

8. Sandra Dial, MD, MSc; J.A.C. Delaney. MSc; Alan N. Narkum, MD, MSc; Samy Suissa, PhD. Use of Gastric Acid-Suppressive Agents and the Risk of Community-Acquired *Clostridium difficile* – Associated Disease. JAMA, 2005; 294: 2989-2995.

9. Sinem Ezgi Gulmez, MD, PhD; Anette Holm, MD, PhD; Henrik Frederiksen, MD, PhD; Thøger Gorm Jensen, MD, PhD; Court Pedersen, DMSC; Jesper Hallas, DMSC. Use of Proton-Pump Inhibitors and the Risk of Community-Acquired Pneumonia. Arch Intern Med. 2007; 167: 950-955.

10. James W. Freston, MD, PhD. Ann Intern Med: August 1994; 121(3); 232-233. omeprazole, Hypergastrinemia, and Gastric Carcinoid Tumors

11. Laura E. Targownik MD MSHS, Lisa M. Lix PhD, Colleen J. Metge OhD, Heather J. Prior MSc, Stella Leung MSc, William D. Leslie MD. Use o Proton Pump Inhibitors and Risk of Osteoporosis-related Fractures. CMAJ 2008; 179(4): 319-26.

19 SCHEDULE OF ASSESSMENTS/STUDY FLOWCHART

19.1 Schedule of Assessments

Assessments	Screening Visit – Visit 1	Randomisation Visit – Visit 2	End of Study Visit – Visit 3
	Day -1 to -7	Day 1	Day 15[1]
Informed Consent/Patient Information	X		
Inclusion/Exclusion Criteria	X	X	
Demographics/Physical Examination	X	X	X
Vital Signs	X	X	
Clinical Laboratory Tests (including follow-up)[2]	X		
General Medical History	X		
Concomitant Medication	X	X	X
Heartburn Severity Score at Specific Time Points following Heartburn Episode[3]	X	X[4]	X[4]
Concomitant Antacids	X	X	X
Pregnancy Test[5]	X		
Adverse Events	X	X	X
E-Diary Use – Instructions/Training[6]	X		
E-Diary – Competent Use/Review	X	X	X

[1] Patient participation may end prematurely if patient experiences 3 episodes of heartburn on 3 separate days before the 14th day
[2] Biochemistry, Haematology and Urinalysis
[3] For 3 episodes of heartburn on 3 separate days
[4] Severity scores recorded at 15, 30, 45, 60, 75, 90, 120, 150 and 180 minutes
[5] For women of child-bearing potential only
[6] Patients will be provided with instruction manuals; Site staff will provide training to all patients

19.2 Study Flowchart

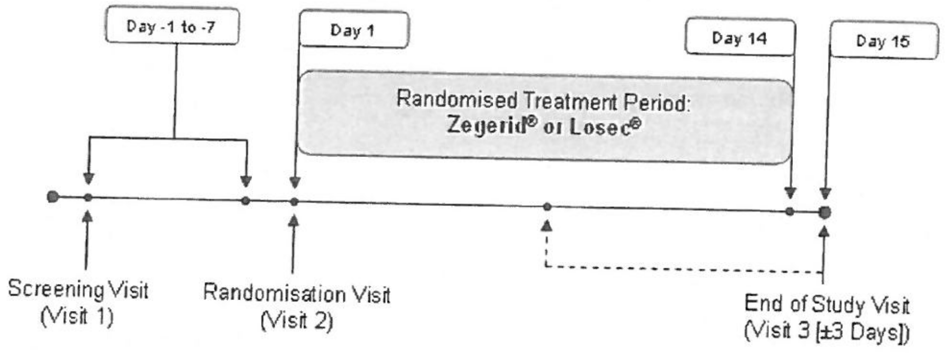

20 APPENDICES

20.1 Declaration of Helsinki:

WORLD MEDICAL ASSOCIATION DECLARATION OF HELSINKI
Ethical Principles for Medical Research Involving Human Subjects

Adopted by the 18th WMA General Assembly, Helsinki, Finland, June 1964, and amended by the:
29th WMA General Assembly, Tokyo, Japan, October 1975
35th WMA General Assembly, Venice, Italy, October 1983
41st WMA General Assembly, Hong Kong, September 1989
48th WMA General Assembly, Somerset West, Republic of South Africa, October 1996
52nd WMA General Assembly, Edinburgh, Scotland, October 2000
53rd WMA General Assembly, Washington 2002 (Note of Clarification on paragraph 29 added)
55th WMA General Assembly, Tokyo 2004 (Note of Clarification on Paragraph 30 added)
59th WMA General Assembly, Seoul, October 2008

A. INTRODUCTION

1. The World Medical Association (WMA) has developed the Declaration of Helsinki as a statement of ethical principles for medical research involving human subjects, including research on identifiable human material and data.

The Declaration is intended to be read as a whole and each of its constituent paragraphs should not be applied without consideration of all other relevant paragraphs.

2. Although the Declaration is addressed primarily to physicians, the WMA encourages other participants in medical research involving human subjects to adopt these principles.

3. It is the duty of the physician to promote and safeguard the health of patients, including those who are involved in medical research. The physician's knowledge and conscience are dedicated to the fulfilment of this duty.

4. The Declaration of Geneva of the WMA binds the physician with the words, "The health of my patient will be my first consideration," and the International Code of Medical Ethics declares that, "A physician shall act in the patient's best interest when

providing medical care."

5. Medical progress is based on research that ultimately must include studies involving human subjects. Populations that are underrepresented in medical research should be provided appropriate access to participation in research.

6. In medical research involving human subjects, the well-being of the individual research subject must take precedence over all other interests.

7. The primary purpose of medical research involving human subjects is to understand the causes, development and effects of diseases and improve preventive, diagnostic and therapeutic interventions (methods, procedures and treatments). Even the best current interventions must be evaluated continually through research for their safety, effectiveness, efficiency, accessibility and quality.

8. In medical practice and in medical research, most interventions involve risks and burdens.

9. Medical research is subject to ethical standards that promote respect for all human subjects and protect their health and rights. Some research populations are particularly vulnerable and need special protection. These include those who cannot give or refuse consent for themselves and those who may be vulnerable to coercion or undue influence.

10. Physicians should consider the ethical, legal and regulatory norms and standards for research involving human subjects in their own countries as well as applicable international norms and standards. No national or international ethical, legal or regulatory requirement should reduce or eliminate any of the protections for research subjects set forth in this Declaration.

B. PRINCIPLES FOR ALL MEDICAL RESEARCH

11. It is the duty of physicians who participate in medical research to protect the life, health, dignity, integrity, right to self-determination, privacy, and confidentiality of personal information of research subjects.

12. Medical research involving human subjects must conform to generally accepted scientific principles, be based on a thorough knowledge of the scientific literature, other relevant sources of information, and adequate laboratory and, as appropriate, animal experimentation. The welfare of animals used for research must be respected.

13. Appropriate caution must be exercised in the conduct of medical research that may harm the environment.

14. The design and performance of each research study involving human subjects must be clearly described in a research protocol. The protocol should contain a statement of the ethical considerations involved and should indicate how the principles in this Declaration have been addressed. The protocol should include information regarding funding, sponsors, institutional affiliations, and other potential conflicts of interest, incentives for subjects and provisions for treating and/or compensating subjects who are harmed as a consequence of participation in the research study. The protocol should describe arrangements for post-study access by study subjects to interventions identified as beneficial in the study or access to other appropriate care or benefits.

15. The research protocol must be submitted for consideration, comment, guidance and approval to a research ethics committee before the study begins. This committee must be independent of the researcher, the sponsor and any other undue influence. It must take into consideration the laws and regulations of the country or countries in which the research is to be performed as well as applicable international norms and standards but these must not be allowed to reduce or eliminate any of the protections for research subjects set forth in this Declaration. The committee must have the right to monitor ongoing studies. The researcher must provide monitoring information to the committee, especially information about any serious adverse events. No change to the protocol may be made without consideration and approval by the committee.

16. Medical research involving human subjects must be conducted only by individuals with the appropriate scientific training and qualifications. Research on patients or healthy volunteers requires the supervision of a competent and appropriately qualified physician or other health care professional. The responsibility for the protection of research subjects must always rest with the physician or other health care professional and never the research subjects, even though they have given consent.

17. Medical research involving a disadvantaged or vulnerable population or community is only justified if the research is responsive to the health needs and priorities of this population or community and if there is a reasonable likelihood that this population or community stands to benefit from the results of the research.

18. Every medical research study involving human subjects must be preceded by careful assessment of predictable risks and burdens to the individuals and communities involved in the research in comparison with foreseeable benefits to them and to other individuals or communities affected by the condition under investigation.

19. Every clinical study must be registered in a publicly accessible database before recruitment of the first subject.

20. Physicians may not participate in a research study involving human subjects unless they are confident that the risks involved have been adequately assessed and can be satisfactorily managed. Physicians must immediately stop a study when the risks are

found to outweigh the potential benefits or when there is conclusive proof of positive and beneficial results.

21. Medical research involving human subjects may only be conducted if the importance of the objective outweighs the inherent risks and burdens to the research subjects.

22. Participation by competent individuals as subjects in medical research must be voluntary. Although it may be appropriate to consult family members or community leaders, no competent individual may be enrolled in a research study unless he or she freely agrees.

23. Every precaution must be taken to protect the privacy of research subjects and the confidentiality of their personal information and to minimize the impact of the study on their physical, mental and social integrity.

24. In medical research involving competent human subjects, each potential subject must be adequately informed of the aims, methods, sources of funding, any possible conflicts of interest, institutional affiliations of the researcher, the anticipated benefits and potential risks of the study and the discomfort it may entail, and any other relevant aspects of the study. The potential subject must be informed of the right to refuse to participate in the study or to withdraw consent to participate at any time without reprisal. Special attention should be given to the specific information needs of individual potential subjects as well as to the methods used to deliver the information. After ensuring that the potential subject has understood the information, the physician or another appropriately qualified individual must then seek the potential subject's freely-given informed consent, preferably in writing. If the consent cannot be expressed in writing, the non-written consent must be formally documented and witnessed.

25. For medical research using identifiable human material or data, physicians must normally seek consent for the collection, analysis, storage and/or reuse. There may be situations where consent would be impossible or impractical to obtain for such research or would pose a threat to the validity of the research. In such situations the research may be done only after consideration and approval of a research ethics committee.

26. When seeking informed consent for participation in a research study the physician should be particularly cautious if the potential subject is in a dependent relationship with the physician or may consent under duress. In such situations the informed consent should be sought by an appropriately qualified individual who is completely independent of this relationship.

27. For a potential research subject who is incompetent, the physician must seek informed consent from the legally authorized representative. These individuals must not be included in a research study that has no likelihood of benefit for them unless it is

intended to promote the health of the population represented by the potential subject, the research cannot instead be performed with competent persons, and the research entails only minimal risk and minimal burden.

28. When a potential research subject who is deemed incompetent is able to give assent to decisions about participation in research, the physician must seek that assent in addition to the consent of the legally authorized representative. The potential subject's dissent should be respected.

29. Research involving subjects who are physically or mentally incapable of giving consent, for example, unconscious patients, may be done only if the physical or mental condition that prevents giving informed consent is a necessary characteristic of the research population. In such circumstances the physician should seek informed consent from the legally authorized representative. If no such representative is available and if the research cannot be delayed, the study may proceed without informed consent provided that the specific reasons for involving subjects with a condition that renders them unable to give informed consent have been stated in the research protocol and the study has been approved by a research ethics committee. Consent to remain in the research should be obtained as soon as possible from the subject or a legally authorized representative.

30. Authors, editors and publishers all have ethical obligations with regard to the publication of the results of research. Authors have a duty to make publicly available the results of their research on human subjects and are accountable for the completeness and accuracy of their reports. They should adhere to accepted guidelines for ethical reporting. Negative and inconclusive as well as positive results should be published or otherwise made publicly available. Sources of funding, institutional affiliations and conflicts of interest should be declared in the publication. Reports of research not in accordance with the principles of this Declaration should not be accepted for publication.

C. ADDITIONAL PRINCIPLES FOR MEDICAL RESEARCH COMBINED WITH MEDICAL CARE

31. The physician may combine medical research with medical care only to the extent that the research is justified by its potential preventive, diagnostic or therapeutic value and if the physician has good reason to believe that participation in the research study will not adversely affect the health of the patients who serve as research subjects.

32. The benefits, risks, burdens and effectiveness of a new intervention must be tested against those of the best current proven intervention, except in the following circumstances:

- The use of placebo, or no treatment, is acceptable in studies where no current proven

intervention exists; or

• Where for compelling and scientifically sound methodological reasons the use of placebo is necessary to determine the efficacy or safety of an intervention and the patients who receive placebo or no treatment will not be subject to any risk of serious or irreversible harm. Extreme care must be taken to avoid abuse of this option.

33. At the conclusion of the study, patients entered into the study are entitled to be informed about the outcome of the study and to share any benefits that result from it, for example, access to interventions identified as beneficial in the study or to other appropriate care or benefits.

34. The physician must fully inform the patient which aspects of the care are related to the research. The refusal of a patient to participate in a study or the patient's decision to withdraw from the study must never interfere with the patient-physician relationship.

35. In the treatment of a patient, where proven interventions do not exist or have been ineffective, the physician, after seeking expert advice, with informed consent from the patient or a legally authorized representative, may use an unproven intervention if in the physician's judgement it offers hope of saving life, re-establishing health or alleviating suffering. Where possible, this intervention should be made the object of research, designed to evaluate its safety and efficacy. In all cases, new information should be recorded and, where appropriate, made publicly available.
