# Supplementary material for: Challenges of Correlating pH Change with Relief of Clinical Symptoms in Gastro Esophageal Reflux Disease: A Phase III, Randomized Study of Zegerid versus Losec
Source: PLoS One. 2015 Feb 23;10(2):e0116308. doi: 10.1371/journal.pone.0116308 (PMC4338010; doi:10.1371/journal.pone.0116308)

STATISTICAL ANALYSIS PLAN

Protocol Title:	A Phase III, Multi-centre, Double-blind, Double-dummy, Randomised, Study to Assess the Superiority of Zegerid® 20 mg vs. Losec® 20 mg in the Rapid Relief of Heartburn Associated with GERD as on Demand Therapy
Protocol Number:	ZEG-01/2010 (GERD) including Amendment 1 (27 July 2011)
Compound:	Zegerid®
Phase:	III
Sponsor:	Norgine Ltd.,<br>Chaplin House,<br>Widewater Place, Moorhall Road,<br>Harefield, Uxbridge, UB9 6NS, UK
SAP Author:	[redacted]<br>Premier Research Group Ltd<br>1st Floor Rubra 2,<br>Mulberry Business Park,<br>Fishponds Road, Wokingham,<br>RG41 2GY, UK
SAP Version:	Final Version v3
SAP Date:	10th November 2011

CONFIDENTIAL

STATISTICAL ANALYSIS PLAN DOCUMENT HISTORY

Version	Date	Author	Description
Final	6th September 2011	[redacted]	Original
Final v2	13th October 2011	[redacted]	Clarification of details
Final v3	10th November 2011	[redacted]	Change to definition of evaluable episode.<br>Additional analyses

SIGNATURE PAGE AND APPROVALS

Dave Walker
Therapy Area Director, Development,
Norgine Ltd

Date

Dr Richard Ng Kwet Shing
Clinical Development Associate Director
Norgine Ltd

Date

[redacted]
Biostatistician
Premier Research Group Ltd

Date

[redacted]
Project Manager,
Premier Research Group Ltd

Date

TABLE OF CONTENTS

1. SYNOPSIS 7
2. STUDY OBJECTIVES AND ENDPOINTS 7
2.1 Study Objectives 7
2.1.1 Primary Objective 7
Secondary Objectives 8
2.2 Efficacy and Safety Endpoints (Target Variables) 8
2.2.1 Efficacy Variables 8
2.2.2 Safety Variables 9
3. STUDY METHODS 10
3.1 Overall Study Design and Plan 10
3.2 Selection of Study Population 11
3.3 Method of Treatment Assignment and Randomisation 11
3.4 Treatment Masking (Blinding) 11
4. ANALYSES AND REPORTING 12
4.1 Interim Analyses 12
4.2 Final Analysis 12
5. SAMPLE SIZE DETERMINATION 12
6. ANALYSIS POPULATIONS 12
6.1 Safety Set 13
6.2 Modified Intention-to-Treat (mITT) Set 13
6.3 Per Protocol (PP) Set 13
7. GENERAL ISSUES FOR STATISTICAL ANALYSIS 13
7.1 General Statistical Methodology 13
7.1.1 Handling of Missing Data 14
7.1.2 Pooling of Centers 15
7.2 Derived and Computed Variables 15
8. STUDY PATIENTS AND DEMOGRAPHICS 16
8.1 Disposition of Patients and Withdrawals 16
8.2 Protocol Violations and Deviations 16
8.3 Demographics and Other Baseline Characteristics 17
9. EFFICACY ANALYSIS 17
9.1 Primary Efficacy Variable Analysis 17
9.2 Secondary Efficacy Variable Analysis 18
10. SAFETY AND TOLERABILITY ANALYSES 19
10.1 Adverse Events 19

10.2 Physical Examination 19
10.3 Concomitant Medication 20
10.4 Treatment Exposure 20

11. REFERENCES 21

12. ATTACHMENTS 21

13. APPENDIX A 22

ABBREVIATIONS

ABBREVIATION	DEFINITION OR DESCRIPTION
AE	Adverse Event
ANOVA	Analysis of variance
ATC	Anatomical Therapeutic Chemical
BMI	Body mass index
CI	Confidence interval
CRF	Case Report Form
CSR	Clinical Study Report
EMA	European Medicines Agency
EU	European Union
FAS	Full Analysis Set
FDA	Food and Drug Administration
GERD	Gastroesophageal reflux disease
ICH	International Conference on Harmonisation
IWRS	Interactive Web Response System
LOCF	Last observation carried forward
LS	Least Squares
MedDRA	Medical Dictionary for Regulatory Activities
mITT	Modified Intention-To-Treat
NTP	Norgine Trial Physician
OTC	Over the Counter
PAR	Protocol adherence report
PP	Per Protocol
PPI	Proton Pump Inhibitor
SAE	Serious Adverse Event
SAP	Statistical Analysis Plan
SD	Standard Deviation
SE	Standard Error
SOC	System Organ Class
TEAE	Treatment-Emergent Adverse Event
WHO	World Health Organisation

1. SYNOPSIS

This Statistical Analysis Plan (SAP) describes the planned analysis and reporting for Norgine protocol ZEG-01/2010 (A Phase III, Multi-centre, Double-blind, Double-dummy, Randomised, Study to Assess the Superiority of Zegerid® 20 mg vs. Losec® 20 mg in the Rapid Relief of Heartburn Associated with GERD as on Demand Therapy), Amendment Version 1.0 Final dated 27th July 2011.

The structure and content of this SAP provides sufficient detail to meet the requirements identified by the Food and Drug Administration (FDA), European Medicines Agency (EMA), and International Conference on Harmonisation (ICH) of Technical Requirements for Registration of Pharmaceuticals for Human Use: Guidance on Statistical Principles in Clinical Trials [1]. All work planned and reported for this SAP will follow internationally accepted guidelines, published by the American Statistical Association [2] and the Royal Statistical Society for statistical practice [3].

The planned analyses identified in this SAP may be included in clinical study reports (CSRs), regulatory submissions, or future manuscripts. Also, post-hoc exploratory analyses not necessarily identified in this SAP may be performed to further examine study data. Any post-hoc, or unplanned, exploratory analyses performed will be clearly identified as such in the final CSR.

The following documents were reviewed in preparation of this SAP:

- Clinical Research Protocol ZEG-01/2010 Amendment Version 1.0 Final issued 27th July 2011.
- Case report forms (CRFs) for Protocol ZEG-01/2010.
- ICH Guidance on Statistical Principles for Clinical Trials (E9).

The reader of this SAP is encouraged to also read the clinical protocol, and other identified documents, for details on the planned conduct of this study. Operational aspects related to collection and timing of planned clinical assessments are not repeated in this SAP unless relevant to the planned analyses.

2. STUDY OBJECTIVES AND ENDPOINTS

2.1 Study Objectives

2.1.1 Primary Objective

The primary objective of this study is to compare Zegerid® 20 mg suspension to Losec® 20 mg capsules with respect to the following: the median time to sustained response, which is defined as a reduction of severity of heartburn associated with gastroesophageal reflux disease (GERD) by 3 points or more on a 9-point Likert severity scale, which is sustained for 45 minutes or more.

Secondary Objectives

Key secondary:

1. Determine the median time to sustained partial response, defined as a reduction of 2 points or more on the 9-point Likert severity scale, which is sustained for 45 minutes or more.
2. Determine the median time to sustained total relief, defined as zero severity (no heartburn) on the 9-point Likert severity scale, which is sustained for 45 minutes or more.
3. Determine the proportion of patients who have achieved sustained response, sustained partial response or sustained total relief by 45, 60 and 90 minutes.

Secondary:

1. Determine the severity of heartburn associated with GERD and change in severity from pre-dose at all time points.
2. Determine the proportion of patients who have achieved sustained response, sustained partial response or sustained total relief at all other time points.
3. Determine the proportion of patients with total relief (defined as zero severity 9-point Likert severity scale) of heartburn associated with GERD at all time points.
4. Determine the proportion of patients with response (defined as a reduction in the severity of heartburn from pre-dose of 3 or more points on the 9-point Likert severity scale) at all time points.
5. Determine the proportion of patients with at least partial response (defined as a reduction in the severity of heartburn from pre-dose of 2 or more points on the 9-point Likert severity scale) at all time points.
6. Determine the area under the severity-time curve (AUC) calculated for the following time periods; 0-60, 0-120 and 0-180 minutes.
7. Determine the usage of Gaviscon® (rescue medication) over the 14 day randomised treatment period.
8. Safety and tolerability.

2.2 Efficacy and Safety Endpoints (Target Variables)

2.2.1 Efficacy Variables

Pain was recorded throughout the data using a 9-point Likert scale (where 0= No heartburn and 8 = Very severe heartburn). The patients recorded their pain score using an electronic diary, the screen used is shown in Appendix A.

The primary efficacy parameter is the time to sustained response, which is defined as a reduction of severity of heartburn associated with GERD by 3 points or more on a 9-point Likert severity scale, which is sustained for 45 minutes or more.

Secondary efficacy variables are:

Key secondary:

1. The time to sustained partial response, defined as a reduction of 2 or more points on the 9-point Likert severity scale, which is sustained for 45 minutes or more.
2. The time to sustained total response, defined as zero severity (no heartburn) on the 9-point Likert severity scale, which is sustained for 45 minutes or more.
3. The proportion of patients who have achieved sustained response, sustained partial relief or sustained total relief by 45, 60 and 90 minutes.

Secondary:

1. The severity of heartburn associated with GERD and change in severity from pre-dose at all time points.
2. The proportion of patients who have achieved sustained response, sustained partial response or sustained total relief at all other time points.
3. The proportion of patients with total relief (defined as zero severity on the 9-point Likert severity scale) of heartburn associated with GERD at all time points.
4. The proportion of patients with response (defined as a reduction in the severity of heartburn from pre-dose of 3 or more points on the 9-point Likert severity scale) at all time points.
5. The proportion of patients with at least partial response (defined as a reduction in the severity of heartburn from pre-dose of 2 or more points on the 9-point Likert severity scale) at all time points.
6. The area under the severity-time curve (AUC) will be calculated for the following time periods; 0-60, 0-120 and 0-180 minutes.
7. The usage of Gaviscon® (rescue medication) over the 14 day randomised treatment period.

2.2.2 Safety Variables

The primary safety parameter is the occurrence of SAEs and AEs. All data will be summarised within each treatment group. All SAEs and AEs will be listed using coding for System Organ Class and Preferred Term (using the MedDRA version 13.1 or higher). Only treatment emergent AEs will be included in the analysis.

Secondary safety parameters are:

- physical examination
- concomitant medication
- rescue medication

3. STUDY METHODS

3.1 Overall Study Design and Plan

This is a multi-centre, double-blind, double-dummy, randomised study to assess the superiority of Zegerid® 20 mg vs. Losec® 20 mg in the rapid relief of heartburn associated with GERD in approximately 194 evaluable patients. Patients are to be recruited in50 study sites in 6 EU countries.

Patients will be randomised to receive either 20 mg Zegerid® suspension plus over-encapsulated placebo capsule or 20 mg Losec® capsule over-encapsulated plus placebo. Patients will also receive Gaviscon® (rescue medication), which will be taken as required for up to 3 tablets (1 tablet is a single dose) per day during the screening period and for up to 2 tablets per day during the randomised treatment period.

Beginning with Screening Visit (Visit 1), patients will start a 1 week baseline symptom assessment period, during which patients will record daily episodes of heartburn using an electronic diary (e-diary) and a 9-point (0-8) Likert severity scale for 7 days. During this time, patients will be requested not to take antacid medication except Gaviscon® (rescue medication), which will be provided by the Investigator.

Patients will receive full instructions and training for use of e-diaries to enable them to record heartburn severity scores. Follow-up assistance will be available through e-diary call centres. The e-diary service provider will issue user and trainer instruction manuals to each site. Staff members involved with the study will provide training to patients at Visit 1 (Screening Visit). Blood samples will also be taken at Visit 1.

Patients will return for Visit 2, 7 days (+2 days) post the Screening Visit when eligibility will be checked as per inclusion criteria, and they will be randomly assigned to one of the two treatment arms. This will be followed by a maximum 14 day period, during which eligible patients will receive one of the two randomly assigned treatments. Patients will be provided with a maximum of 3 days of randomised study medication and will be instructed to self-administer 1 pack of randomised study medication (1 dose each of suspension and capsule) immediately when they experience an episode of heartburn (maximum 1 pack per day for a total of 3 days out of 14 days) from Day 1. Patients can continue to take rescue medication on Day 0 (Visit 2).

From Day 1, between 06:00 and 22:00 hours, if a patient experiences an episode of heartburn, for which they feel that taking medication is necessary, they will be required to immediately take their randomised study medication (capsule and suspension) and to record the severity of the heartburn episode (pre-dose) using an e-diary as provided. Patients will be requested to grade the severity of the heartburn at 15, 30, 45, 60, 75, 90, 105, 120, 150 and 180 minutes after taking the randomised study medication (severity scores will be recorded for a total of 3 hours) using their e-diary.

If the patient experiences more than 1 episode in a day then they should use Gaviscon® (rescue medication) as provided for up to a maximum of 2 tablets per day but not within

the initial 3 hours after dosing with randomised study medication.
After 3 episodes of heartburn on 3 separate days where randomised study medication has been taken and recorded on e-diaries, patients should contact the Investigator. Patients will participate for a maximum of 14 days post randomisation to ensure 3 days of heartburn treatment are recorded, however patient participation may end prematurely if 3 episodes of heartburn on 3 separate days are recorded before the 14th day. Patients can continue to take rescue medication until they see the Investigator.

Patient reported outcomes will be recorded using the e-diary by the patients when they have an episode of heartburn and have taken randomised study medication.

1. Severity of heartburn at time zero just prior to randomised study medication being taken.
2. The severity of heartburn recorded at 15, 30, 45, 60, 75, 90, 105, 120, 150 and 180 minutes after the start of randomised study medication for each episode.

3.2 Selection of Study Population

Approximately 300 patients with a history of GERD who have completed PPI therapy and are previously symptomatically responsive (with an improvement of symptom on PPI) to PPI therapy standard dose and continue to experience heartburn 2-3 days per week, will be enrolled to obtain 194 evaluable patients.

3.3 Method of Treatment Assignment and Randomisation

Patients are to be randomised to treatment if they satisfy all the inclusion criteria and are not precluded from participation by any of the exclusion criteria. Patients must also comply with screening period requirements.

Each patient will receive a unique patient number at the Visit 1 (Screening Visit). If the patient is found to be eligible for study participation at the Visit 2 (Randomisation Visit) he/she will be assigned a random number and will receive the study medication with the corresponding number. In case of a withdrawal, the patient's random number will not be reassigned to another patient.

The randomisation list will be produced by a statistician of Premier Research Group Ltd. who is not involved in the conduct or analysis of the study using the SAS system for Windows (SAS Institute Inc, Cary, North Carolina, United States of America [USA]). The randomisation schedule will then be made available to Premier Research IWRS (Interactive Web Response System) and sent to Norgine Ltd. drug packaging group.

3.4 Treatment Masking (Blinding)

This is a double-blind, double-dummy randomised study with both Zegerid® and Losec® being provided in the same packaging and labelling in order to maintain blinding.

No person involved in conducting the study will have access to the randomisation code before the blind is officially broken. However, in the case of an emergency that requires the investigator to be unblinded, the investigator can obtain the randomisation code via

Premier Research IWRS. Only users with specified access via a user identification and password assigned by Premier Research will be able to unblind the patient. The Norgine Drug Safety Group representative will also have specified access and can determine which treatment for a particular patient was given by accessing Premier Research IWRS. The Investigator or Norgine Ltd. will state the reason why the code was broken as part of the IWRS process.

4. ANALYSES AND REPORTING

4.1 Interim Analyses

There are no interim analyses planned for this study.

4.2 Final Analysis

All final, planned, analyses identified in the protocol and in this SAP will be performed only after all patients have completed the Day 15 visit and all AEs have been resolved or followed-up for 4 weeks. In addition, no database may be locked or analyses completed, until this SAP has been approved.

Key statistics and study results will be made available to Norgine following database lock and prior to completion of the final CSR.

Any post-hoc, exploratory analyses completed to support planned study analyses, which were not identified in this SAP, will be documented and reported in appendices to the CSR. Any results from these unplanned analyses (post-hoc) will also be clearly identified in the text of the CSR.

5. SAMPLE SIZE DETERMINATION

Zegerid® has an expected median time to response of 45 minutes compared to a median time to response for Losec® of 75 minutes. With 90 % power and a two-sided level of significance of 0.05 (5%), 97 patients are required per treatment group.

The software nQuery Advisor v6.1 was used for the calculation and a log-rank test assumed.

6. ANALYSIS POPULATIONS

Decisions regarding data exclusion from each population will be taken in consultation with Norgine Ltd. prior to breaking the randomisation code (blind review) and will be documented in the Protocol Adherence Report (PAR) which will be agreed and signed after the review and prior to unblinding.

6.1 Safety Set

The Safety Set will consist of all randomised patients who received at least one dose of randomised study medication during the course of the study.

6.2 Modified Intention-to-Treat (mITT) Set

The Modified Intention-To-Treat (mITT) population will consist of all patients in the Safety population who are evaluable (recorded data for at least one evaluable episode of heartburn).

6.3 Per Protocol (PP) Set

The Per Protocol (PP) population will consist of all patients included in the mITT population but will exclude the following:

1. Patients violating any inclusion/exclusion criterion
2. Patients with major protocol violations (e.g., poor compliance, incorrect completion of e-diaries) in a sufficiently serious manner to warrant exclusion.

7. GENERAL ISSUES FOR STATISTICAL ANALYSIS

7.1 General Statistical Methodology

Descriptive summaries will be provided where appropriate for each of the primary and secondary variables. In general, summaries will be presented by patient population and by treatment groups and/or overall. Where data are collected over time, both the observed data and the change from the run-in period will be summarised at each time point.

In general, continuous, quantitative, variable summaries will include the number of patients (N) (with non-missing values), mean, standard deviation (SD), median, minimum and maximum, 1st and 3rd quartiles, and the number of missing values, as appropriate.

The minimum and maximum statistics will be presented to the same number of decimal places as the original data. The mean, LS mean and median will be presented to one more decimal place than the original data. The SD and SE will be presented to two more decimal places than the original data. When summaries of changes from baseline are presented, the tables will also include a summary of baseline values as well.

Categorical, qualitative, variable summaries will include the frequency and percentage of patients who are in the particular category. In general the denominator for the percentage calculation will be based upon the total number of patients in the study population for the treatment group and/or overall, unless otherwise specified.

All CRF and non-CRF data collected will be presented within data listings. The data listings will in general be sorted by treatment group followed by unrandomised patients. Within these groups they will be sorted by site number and patient number and presented chronologically.

The patient groups in the tables, listings and figures will be identified by labels describing the treatment, e.g., Zegerid or Losec.

All hypothesis testing will be carried out at the 5% (2-sided) significance level. Should any of the statistical methods proposed prove unsuitable during the final analysis, alternative methods will be considered and any changes documented in the clinical study report (CSR), including the rationale for use.

7.1.1 Handling of Missing Data

Heartburn Measurements

An episode is not evaluable if data are missing from more than 1 time point in the 3 hour period.

If there is one timepoint with missing data, the value will be estimated using linear interpolation from the data on either side.

Prior/Concomitant Medication

If the start/stop date of medication is partially or completely missing a medication will be assumed to be concomitant if it cannot be definitely shown that the medication was not administered during the treatment period. Missing dates will not be replaced.

Thus, the following approach will be taken for exclusion from concomitant medication because of discontinuation before start of treatment:

- If stop day is missing but month is complete, medication will only be excluded from concomitant medication if stop month is before month of treatment start.
- If stop day and month are missing but year is complete, medication will only be excluded from concomitant medication if stop year is before year of treatment start.
- If stop date is completely missing, medication will not be excluded.

Adverse Events

If the start date of an AE is partially or completely missing, the AE will be assumed to be treatment-emergent if it cannot be definitely shown that the AE did not occur or worsen during the treatment emergent period (worst case approach). Missing dates will not be replaced.

Thus, the following approach will be taken:

- If the start date is complete, an AE will only be excluded from treatment emergent AEs if the start day is before the day of first treatment, or the start day is after the end day of the treatment emergent period.
- If the start day is missing but the start month is complete, an AE will only be excluded from treatment emergent AEs if the start month is before the month of first treatment or the start month is after the end month of the treatment emergent period, or if the stop date is before the start of first treatment.
- If the start day and months are missing but the start year is complete, an AE will only be excluded from treatment emergent AEs if the start year is before the year

of first treatment or if the start year is after the end year of the treatment emergent period, or if the stop date is before the start of first treatment.

- If start date is completely missing, an AE will not be excluded from treatment-emergent AEs unless the stop date is before the start of first treatment.

7.1.2 Pooling of Centers

The study will be performed in approximately 50 study sites in 6 EU countries. The sites will be combined into countries for inclusion in the analysis.

7.2 Derived and Computed Variables

The following derived and computed variables have been initially identified as important for the analysis of the primary and secondary target variables. It is expected that additional variables will be required. The SAP will not be amended for additional variables that are not related to the primary target or key secondary target variables. Any additional derived or computed variables will be identified and documented in the SAS programs that create the analysis files. If the SAP is not amended, further derivations related to primary and secondary target variables will be described in the CSR.

Each patient will have data from between 1 and 3 episodes of heartburn that have been treated with the study medication. The median will be used to give an average severity of heartburn for each patient at each nominal time point and this will be used for all analyses.

An evaluable episode will be one with a baseline severity of level 4 (point 3 on a 0-8 point scale) or more, no more than 1 missing time point in the 3 hour period and where no rescue medication had been taken in the 4 hours prior to taking the study medication.

The following will be derived for each patient:

Time to Sustained Response:
The start of the period where the pain score is reduced by 3 points or more on a 9-point Likert severity scale from pre-dose and remains at that level (or better) for 45 minutes or more. The times of the start and end of the period will be calculated using interpolation if necessary (for example if the first fall in the pain score is 4 or more).

Time to Sustained Partial Response:
The start of the period where the pain score is reduced by 2 points or more on a 9-point Likert severity scale from pre-dose and remains at that level (or better) for 45 minutes or more. The times of the start and end of the period will be calculated using interpolation if necessary.

Time to Sustained Total Relief:
The start of the period where the pain score is 0 on a 9-point Likert severity scale and remains at that level (or better) for 45 minutes or more.

Response
A patient will be classified as having either sustained response, sustained partial response or sustained total response.

Additionally, at each timepoint a patient will be classified as having either response (a reduction of 3 points from pre-dose), partial response (a reduction of 2 points from pre-dose), or total response (a pain score of 0). The best response will be derived.

AUC of severity
The area under the severity-time curve (AUC) will be calculated using the trapezoidal rule and the nominal times for 3 periods: 0-60, 0-120 and 0-180 minutes. Where data are missing from the end of a period, the last observation carried forward (LOCF) method will be used.

Rescue Medication
The usage of Gaviscon® (rescue medication) over the 14 day randomised treatment period will be calculated using the difference between the number of tablets of rescue medication dispensed on Day 0 and the number returned at the end of the study. The number of tablets taken per day will be derived, using the period from Day 1 to the last day with data in the ediary.

8. STUDY PATIENTS AND DEMOGRAPHICS

8.1 Disposition of Patients and Withdrawals

All patients who provided informed consent are to be accounted for in this study. Descriptive summaries of population data will include:

- the frequency and percent of patients in each study population by centre/country,
- the disposition of patients (including number of patients enrolled, number of patients randomised, number of completers),
- study withdrawals by reason of withdrawal

and will be presented for all enrolled patients by treatment group and overall.

8.2 Protocol Violations and Deviations

Protocol violations will be checked on complete data for all patients prior to defining the analysis populations. The final decision regarding inclusion/exclusion of patients from the analysis sets will be taken during a blinded Data Review Meeting before data hardlock and unblinding and the final analysis. The decision will be based on the blinded raw data listings and the protocol violations and deviations tracked by Project Management. A major protocol violation will lead to an exclusion from the PP Population; minor violations are acceptable.

Individual patients with any protocol deviation will be listed. Major protocol violations will be summarised by type of violation and by treatment group and overall.

8.3 Demographics and Other Baseline Characteristics

Descriptive summaries of the demographic and other baseline characteristics will be completed for the populations specified below by treatment group and overall, unless otherwise specified.

Descriptive summaries of demographic and other baseline conditions will include:

- Demographics (Age, Gender, Race, Height, Weight, Body Mass Index [BMI]) (Safety, mITT, and PP Populations)
- Medical History (Safety Population)
- Baseline Vital Signs (Safety Population)
- Prior Medications (Safety Population)
- Baseline Laboratory Screening including urinalysis, haematology, biochemistry and pregnancy test (Safety Population)

Medical History: Note that medical history will be coded with Medical Dictionary for Regulatory Activities (MedDRA Version 13.1). Incidences of findings in medical history will be summarised by system organ class (SOC) and preferred term and treatment group.

Baseline physical examination: Any abnormalities seen at screening are recorded as medical history and therefore are not analysed separately

Prior medication: Note that all medications will be coded using the World Health Organisation (WHO) Drug Dictionary (September 2010). Incidences of prior medications will be summarised by Anatomical Therapeutic Chemical (ATC) level 2 and ATC level 4 by treatment group.

Prior medication is defined as any medication that was administered prior to the start of the treatment period. The treatment period is defined as the time from day of first treatment to the End of Study visit (scheduled for Day 15).

Laboratory Screening: The CRF records whether the results of each test are clinically significant or not clinically significant, the data will be listed and summarised in frequency tables.

9. EFFICACY ANALYSIS

All efficacy analyses will use the mITT and the PP sets.

9.1 Primary Efficacy Variable Analysis

The primary efficacy parameter is the time to sustained response, which is defined a reduction of 3 points or more on the 9-point Likert severity scale which is sustained for 45 minutes or more. This will be defined using the average (median) heartburn severity at each time point. Patients who do not achieve sustained response will be censored at the last evaluable time point. The time to the start of this response will be shown in each treatment group using a Kaplan-Meier curve. The median time and the 95% confidence interval will be presented for each treatment. Differences between the two treatments will be tested at a two-sided significance level of 0.05 (5%) using a Cox regression model, including a country effect. A 95% confidence interval will be presented for the treatment

hazards ratio.

The primary statistical hypothesis to be tested is that there is no difference in the time to sustained relief in the two treatment groups:

H_0: Median time in Zegerid group / Median time in Losec group = 1
H_1: Median time in Zegerid group / Median time in Losec group ≠ 1

9.2 Secondary Efficacy Variable Analysis

Key Secondary efficacy parameters:
The time to sustained partial response and the time to sustained total relief will be analysed in the same way as the primary efficacy parameter.

The cumulative proportion of patients who have achieved sustained response, sustained partial response or sustained total relief in each treatment group within 45, 60 and 90 minutes will be presented. A 95% confidence interval will be represented for the treatment difference at each time point along with the probability of a difference between treatment groups (chi-squared test).

Secondary efficacy parameters:
The severity of heartburn, change from baseline and percentage change from baseline will be summarised with descriptive statistics for each treatment at each time point. The mean severity, change and percentage change at each timepoint will be shown graphically.

The cumulative proportion of patients who have achieved sustained response, sustained partial response or sustained total relief in each treatment group at all other time points will be presented.

The proportion of patients who have achieved response, partial response or total relief in each treatment group will be summarised at all time points by treatment in a frequency table.

The best sustained response and the best response will be summarised for each treatment.

The AUCs will be summarised with descriptive statistics for each treatment.

The usage of Gaviscon® (rescue medication) over the screening and the randomised treatment period will be summarised as the average number of tablets taken per day by treatment.

The number of hours between taking the study medication and the next intake of Gaviscon® (rescue medication) will be derived and summarised for each treatment.

No testing is planned for any of these secondary parameters, although further exploratory analyses may be added. These will be described in the Clinical Study Report.

10. SAFETY AND TOLERABILITY ANALYSES

The analysis of safety assessments in this study will include summaries of the following categories of safety and tolerability data collected for each patient and will be presented for the Safety Population

10.1 Adverse Events

The primary safety parameter is the occurrence of SAEs and AEs. All data will be summarised within each treatment group. All SAEs and AEs will be listed using coding for System Organ Class and Preferred Term (using the MedDRA version 13.1).

Only treatment-emergent AEs (TEAE) will be included in the analysis. Treatment emergent AEs are defined as AEs occurring or worsening after the start date of the first study treatment and up to 28 days after the last dose of study treatment (treatment emergent period). However, any AE occurring or worsening defined as treatment emergent if these are considered to be related to the study drug.

Time from first treatment to onset of AE (days) will be calculated for complete dates only and will be included in listings.

An AE summary table will be presented including rows with the number of patients with

- All treatment-emergent AEs
- Serious Adverse Events (SAEs)
- Drug-related TEAEs
- Serious Drug-Related TEAEs
- TEAEs leading to discontinuation of study drug
- TEAEs leading to death

AEs will be summarised as follows:

- Number and percentage of patients with AEs classified by System Organ Class and Preferred Term
- Number and percentage of patients by strongest relationship to randomised study medication, System Organ Class and Preferred Term
- Number and percentage of patients by maximum severity, System Organ Class and Preferred Term
- Number and percentage of patients with serious adverse events (SAEs) classified by System Organ Class and Preferred Term
- Number and percentage of patients with serious drug-related adverse events (SAEs) classified by System Organ Class and Preferred Term

A data listing of AEs leading to withdrawal of study drug will be provided, displaying details of the event(s) captured on the CRF.

10.2 Physical Examination

Any abnormalities seen during the study are recorded as adverse events and therefore not analysed separately.

10.3 Concomitant Medication

Incidences of concomitant medications will be summarised by Anatomical Therapeutic Chemical (ATC) level 2 and ATC level 4 by treatment group.

Concomitant medication is defined as a medication other than study drug that was administered during the treatment period. The treatment period is defined as the time from day of first treatment to the End of Study visit (scheduled for Day 15). Previous medication which was discontinued before start of treatment (i.e., prior medication) and medication which was started after the treatment period will be excluded from concomitant medication.

10.4 Treatment Exposure

The total number of doses taken will be calculated. Descriptive statistics will be presented by treatment group.

11. REFERENCES

1. US Federal Register. (1998) International Conference on Harmonization; Guidance on Statistical Principles for Clinical Trials. Department of Health and Human Services: Food and Drug Administration [Docket No. 97D-0174]. Federal Register Volume 63, Number 179, pages 49583-49598. September 16, 1998.
2. ASA. (1999) Ethical Guidelines for Statistical Practice. Prepared by the Committee on Professional Ethics, August 7, 1999.
3. RSS. (1993) The Royal Statistical Society: Code of Conduct, August 1993.

12. ATTACHMENTS

The Table of Contents for the Tables, Listings and Figures along with table shells is given in a separate document:

Norgine GERD SAP TLGs FINAL v3_10NOV2011.doc

13. APPENDIX A

Likert Scale as presented in the eDiary:

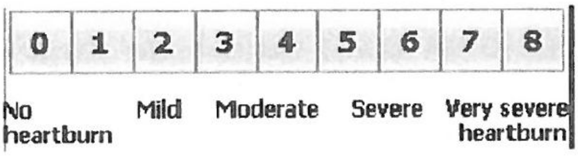

Supplement: S1 File — (PDF) [file pone.0116308.s003.pdf]
